# Selected Compounds Structurally Related to Acyclic Sesquiterpenoids and Their Antibacterial and Cytotoxic Activity

**DOI:** 10.3390/molecules200611272

**Published:** 2015-06-18

**Authors:** Radosław Bonikowski, Paulina Świtakowska, Monika Sienkiewicz, Małgorzata Zakłos-Szyda

**Affiliations:** 1 Institute of General Food Chemistry, Łódź University of Technology, Stefanowskiego St. 4/10, 90-924 Łódź, Poland; E-Mail: paulina@toya.net.pl; 2 Environmental Biology Department, Medical University of Łódź, Żeligowskiego St. 7/9, 90-752 Łódź, Poland; E-Mail: monika.sienkiewicz@umed.lodz.pl; 3 Institute of Technical Biochemistry, Łódź University of Technology, Stefanowskiego St. 4/10, 90-924 Łódź, Poland; E-Mail: malgorzata.zaklos-szyda@p.lodz.pl

**Keywords:** terpenoids, geranyl acetone, nerolidol, farnesol, farnesyl acetate, structurally related compound, antimicrobial activity, cytotoxicity

## Abstract

By implementing a common and industrially used method, 30 compounds which are structurally related to geranyl acetone, nerolidol, farnesal, farnesol and farnesyl acetate were obtained. Their antimicrobial activity against *Staphylococcus aureus*, *Enterococcus faecalis*, *Enterococcus faecium*, *Escherichia coli*, *Klebsiella pneumoniae* and *Acinetobacter baumannii* bacteria was investigated. Pharmacophore models were proposed based on the obtained results and 3D QSAR modelling. Cytotoxic effects against mainly human immortalised and normal cell lines of different origin (malignant melanoma MeWo, colorectal adenocarcinoma HT29, promyelocytic leukemia HL60, gingival fibroblasts HFIG, skin keratinocytes HaCaT and rat small intestine epithelium IEC6) were examined. The odour descriptions of newly synthesised compounds are given.

## 1. Introduction

**Figure 1:**
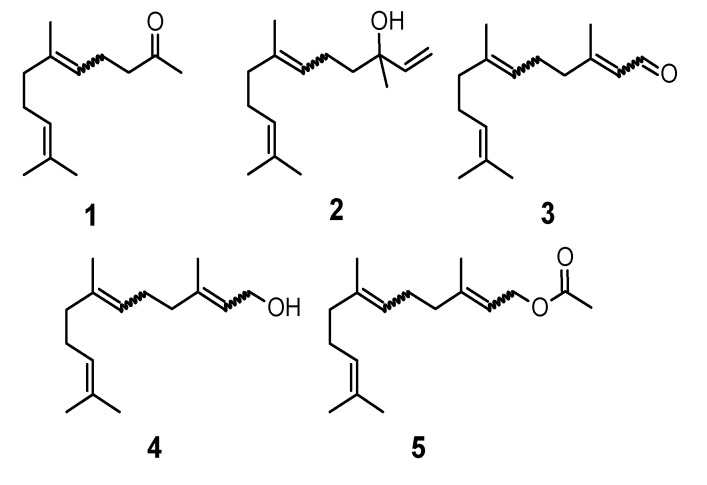
Structures of geranyl acetone (**1**); nerolidol (**2**); farnesal (**3**); farnesol (**4**) and farnesyl acetate (**5**).

Most of the natural acyclic sesquiterpenoids are used in the fragrance and flavour industry as odorants and flavourings. This group includes geranyl acetone (**1**), nerolidol (**2**), farnesal (**3**), farnesol (**4**) and farnesyl acetate (**5**) (Figure 1).

Nerolidol and farnesol, in addition to their flavouring characteristics, also possess antibacterial properties. Farnesol, which is present in many creams and nourishing agents, has a bacteriostatic effect against bacteria that can cause acne (*Staphylococcus aureus*, *Staphylococcus epidermidis*) and does not affect naturally occurring microflora on the skin [1]. It blocks the formation of biofilm by the drug-resistant strains of *Staphylococcus aureus* and acts synergistically with antibiotics [2,3,4]. Brilhante *et al.* showed that farnesol had an inhibiting activity alone and in combination with antifungal agents against strains of dimorphic fungi from the genus *Coccidioides* and against *Candida* spp. clinical isolates [4,5]. Nerolidol shows a toxic effect against, *inter alia*, *Salmonella enterica*, *Staphylococcus aureus*, *Escherichia coli* and *Aspergillus niger*, and increases the antibacterial activity of antibiotics [6,7]. Nagaki *et al.* examined farnesal activity against *S. aureus* finding that it showed the highest activity among the analysed terpenoids [8]. Shin *et al.* evaluated antimicrobial activity of farnesyl acetate which inhibited the growth of *S. aureus* and *Pseudomonas aeruginosa* [9]. Geranyl acetone has also antimicrobial properties as shown by the examination of its food preservative ability [10]. Moreover, natural sesquiterpenoids *in vivo* and *in vitro* have chemopreventive and chemotherapeutic properties against human cancer cell lines (inter alia: human malignant melanoma, promyelocytic leukemia and lung cancer) [11,12,13,14].

In our previous studies [15,16,17], we described the synthesis and mainly the odour activity of the analogues of acyclic terpenoids where isobutenyl moiety was replaced by a furan or thiophene ring.

**Figure 2:**
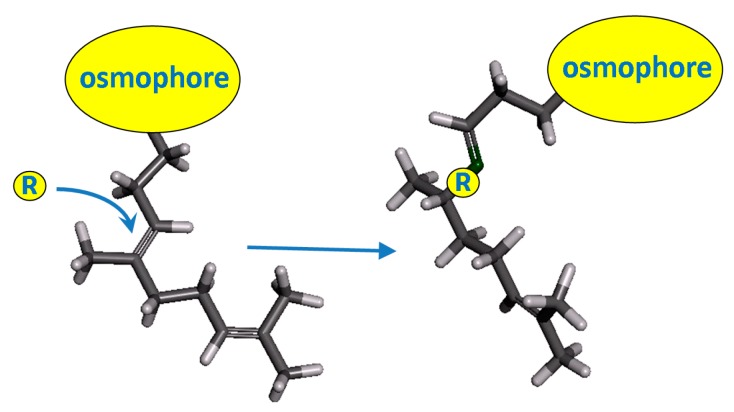
The structural relationship of the examined compounds to the natural sesquiterpenoids.

This time we decided to examine the bioactivity (scent, antibacterial and cytotoxic) of compounds structurally related to acyclic sesquiterpenoids where the osmophore and the 6-methylheptenone moiety will be maintained, and the changes will be introduced into the middle part of the chain as shown in Figure 2.

**Figure 3:**
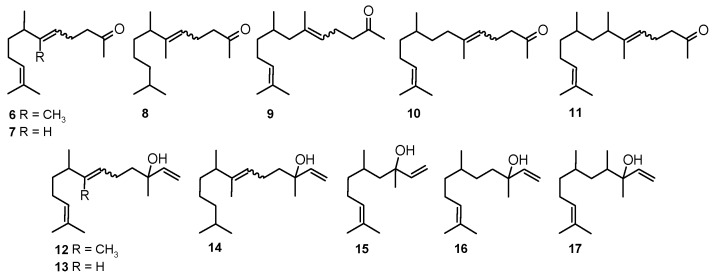
Compounds structurally related to geranyl acetone (**6** to **11**) and nerolidol (**12** to **17**).

We reported compounds structurally related to geranyl acetone and nerolidol (Figure 3) before [18] due to their olfactory and antibacterial properties as potential fragrance components which, according to current trends, should possess added values, especially preserving properties, thus allowing for a reduction or elimination of traditional cosmetic preservatives [19,20].

## 2. Results and Discussion

### 2.1. Synthetic Methodology

**Figure 4:**
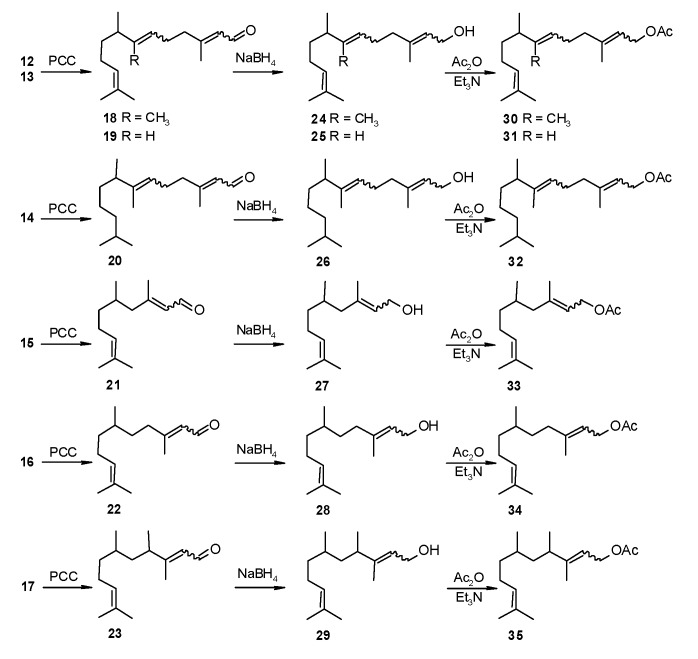
Synthesis of compounds structurally related to farnesal (**18** to **23**), farnesol (**24** to **29**) and farnesyl acetate (**30** to **35**).

Starting from the alcohols **12**–**17** [18], we obtained a series of compounds structurally related to farnesal (**18**–**23**) (Figure 4) by an oxidative rearrangement of tertiary allylic alcohols [21].

We observed that the obtained aldehydes exhibited high lability, which could somehow be confirmed by the dependence of the yield on the isolation method. The yields of the distilled products were 36% to 67%, while for products purified by column chromatography on silica gel, the yields were 71% to 83%. Furthermore, we found that the aldehydes **18**–**23** were rather stable in the solutions. The *E*/*Z* geometric isomer ratio, confirmed by GC and NMR analyses, of the rearranged C-C double bond was 2:1 for **18**, **19**, **20**, **23**, while for **21** and **22** it was 1:1 and 3:2, respectively. The most characteristic and diagnostic signal on the NMR spectra was the signal of the γ methyl substituent. In the case of the *E* isomer, the ^1^H chemical shift, due to deshielding by the carbonyl group, was higher (*ca.* 2.1 ppm and *ca.* 1.9 ppm for the *Z* isomer). On the other hand, in the *E* isomer, the signal of the carbon atom, due to the steric effect, was shifted upfield (*ca.* 17 ppm and *ca.* 25 ppm for the *Z* isomer).

The scent of the compounds structurally related to farnesal, or rather its absence, was surprising. The only exception was the odour of **21**, which was intense, floral with fatty and melon notes, and of **23**, which was geranyl acetone-like (green, fruity, waxy and woody).

The obtained aldehydes reduced with sodium borohydride in the usual manner yielded compounds structurally related to farnesol (47% to 97%). The scent of the products was faint and floral, except for **29**, which was odourless.

Subsequent esterification with acetic anhydride in the presence of triethylamine gave compounds structurally related to farnesyl acetate (yield 69% to 90%) which, just as the compounds structurally related to farnesal, were generally odourless but with one exception. Compound **35** had a very faint quince-like scent.

For all compounds which had the scent, we performed additionally GC-olfactometry experiments. For the compounds structurally related to both geranyl acetone and nerolidol [18], we did not observe significant differences in the odour description of the isomers.

### 2.2. Antibacterial Activity of the Terpenoids and Their Structurally Related Compounds

All of the obtained compounds and the parent terpenes were subjected to an evaluation of their antibacterial activity (Table 1). Data for geranyl acetone, nerolidol, ketones **6** to **11** and alcohols **12** to **17** were presented by us before [18].

Generally, the ketones **6** to **11** demonstrated the highest antibacterial activity against both standard strains from the *Enterococcus* genus and all of the tested Gram-negative bacteria with a Minimal Inhibitory Concentrations (MIC) between 12 µg/mL and 32 µg/mL. Parent terpenoid-farnesol and some alcohols related to it and their acetates showed slightly similar activity. The most resistant to all of the tested compounds was the standard strain of *Staphylococcus aureus* ATCC 43300. Although Togashi *et al.* found that farnesol was active against *Staphylococcus aureus* FDA209P at concentrations of 10 μg/mL and higher, in their investigations the bacterial suspension contained 1 × 10^5^ CFU and the culture was incubated for 48 h with shaking and monitored turbidimetrically [2]. On the other hand, Jin *et al.* stated that farnesol had an inhibiting activity against *Mycobacterium smegmatis* mc^2^ 155 ATCC 700084 with a MIC value at 64 µg/mL by micro-dilution and in accordance with NCCLS guidelines [22]. The MIC values obtained in our research for farnesol were from 16 µg/mL to 52 µg/mL against all of the tested standard strains. The other tested terpenoids and their structurally related compounds had an inhibiting activity against standard bacteria at a concentration between 12 µg/mL and 54 µg/mL.

For synthesised compounds we also obtained by column chromatography (*E*)-(*Z*)-isomer enriched fractions containing ≥ 85% of the appropriate isomer according to the GC, but the determined bioactivity (antimicrobial and cytotoxicity) did not differ significantly from that obtained for the mixtures (data not presented). Ciproflaxicin and gentamicin were used as the control for the reference strains.

**Table 1:** Antibacterial activity of structurally related compounds and parent terpenes against Gram-positive and Gramnegative reference strains.

Compound	Minimum Inhibitory Concentrations MIC (µg/mL)					
	Gram-Positive Bacteria			Gram-Negative Bacteria		
	*Staphylococcus aureus* ATCC 43300	*Enterococcus faecalis* ATCC 51299	*Enterococcus faecium* ATCC 35667	*Escherichia coli* ATCC 25922	*Klebsiella pneumoniae* ATCC 700603	*Acinetobacter baumannii* ATCC 19606
Geranyl acetone (**1**)	48	36	28	26	18	18
**6**	26	18	16	14	14	12
**7**	26	16	18	16	16	14
**8**	38	26	28	18	18	22
**9**	28	18	14	28	18	16
**10**	46	26	18	32	18	16
**11**	26	22	18	28	28	14
Nerolidol (**2**)	48	38	32	46	16	26
**12**	48	46	42	40	28	26
**13**	46	42	38	40	20	22
**14**	48	38	36	38	18	28
**15**	46	48	38	28	16	16
**16**	48	38	36	32	24	22
**17**	48	38	38	46	18	16
Farnesal (**3**)	24	22	36	46	44	36
**18**	18	22	28	52	46	46
**19**	28	34	38	44	36	42
**20**	22	20	32	48	38	38
**21**	32	38	42	54	48	52
**22**	26	24	46	44	36	36
**23**	28	26	36	46	34	36
Farnesol (**4**)	26	18	16	28	16	18
**24**	22	18	32	52	44	40
**25**	28	32	34	50	48	44
**26**	32	30	36	48	42	38
**27**	28	26	18	36	18	34
**28**	26	18	16	28	16	18
**29**	28	22	18	38	36	32
Farnesyl acetate (**5**)	28	20	20	28	22	22
**30**	30	24	18	30	24	24
**31**	24	20	20	26	22	18
**32**	28	22	20	28	18	22
**33**	26	18	16	26	18	22
**34**	32	20	18	18	20	18
**35**	34	16	14	16	18	12
Ciprofloxacin	8	2	8	4	2	4
Gentamicin	16	>64 *	32	4	32	32

* The maximum examined concentration was 64 µg/mL.

#### Pharmacophores

**Figure 5:**
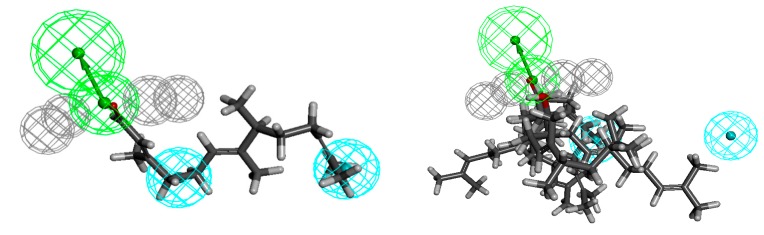
Aldehyde **18** aligned to the pharmacophore model of *Staphylococcus aureus* active compounds (on the left), the least active ones **1**, **2**, **12**, **14**, **16** and **17** (on the right).

**Figure 6:**
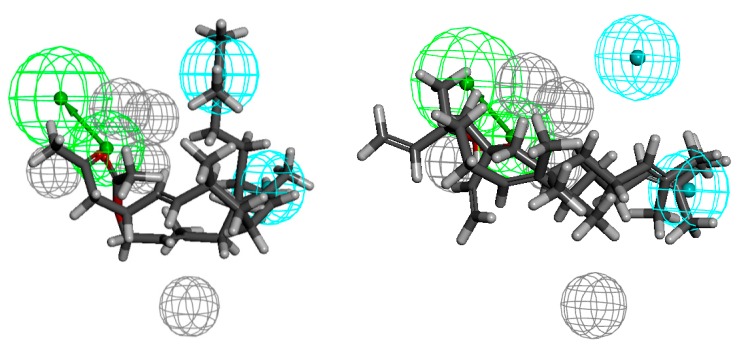
Compounds structurally related to geranyl acetone and farnesyl acetate (**7** and **35**) aligned to the pharmacophore model of *Enterococcus faecalis* active compounds (on the left), the least active ones **12** and **15** (on the right).

**Figure 7:**
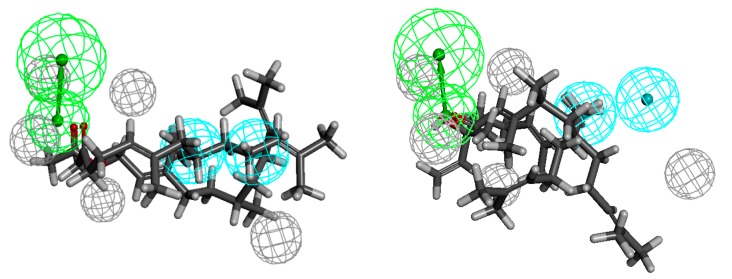
Compounds structurally related to geranyl acetone and farnesyl acetate **9** and **35** aligned to the pharmacophore model of *Enterococcus faecium* active compounds (on the left), the least active ones **12** and **22** (on the right).

**Figure 8:**
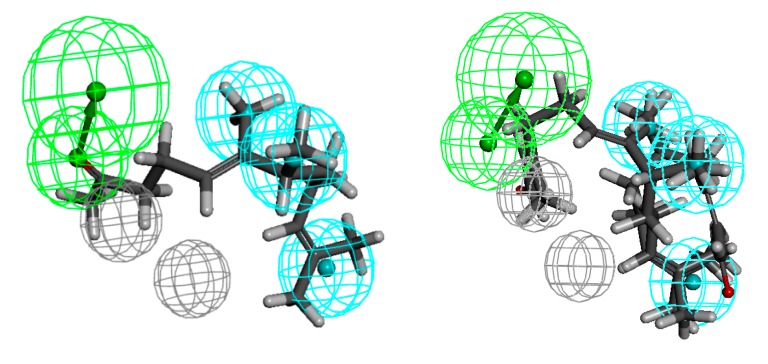
Compound structurally related to geranyl acetone **6** aligned to the pharmacophore model of *Escherichia coli* active compounds (on the left), the least active ones **18** and **21** (on the right).

**Figure 9:**
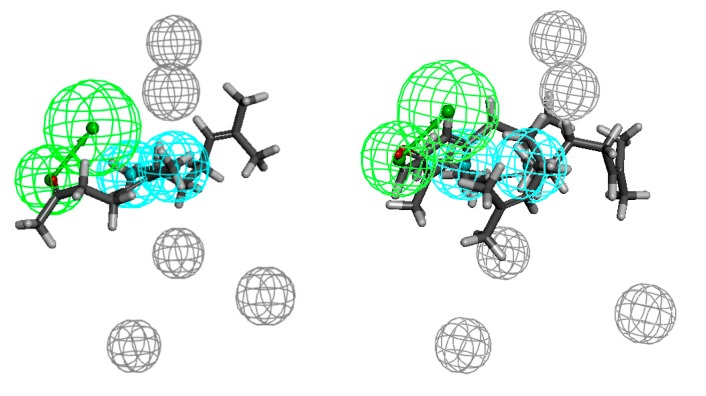
Ketone **6** aligned to the pharmacophore model of *Klebsiella pneumoniae* active compounds (on the left), the least active ones **18** and **21** (on the right).

**Figure 10:**
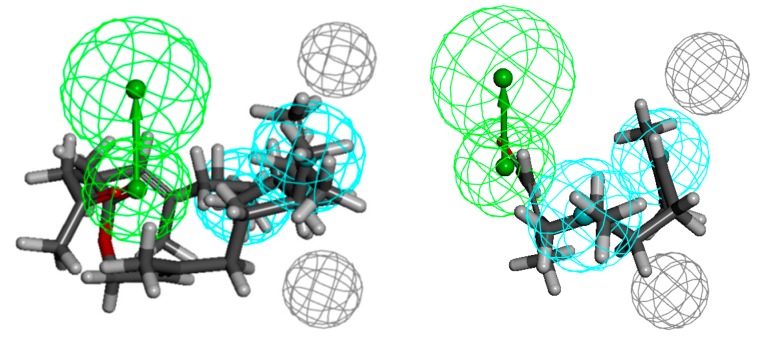
Compounds structurally related to geranyl acetone and farnesyl acetate **6** and **35** aligned to the pharmacophore model of *Acinetobacter baumannii* active compounds (on the left), the least active ones **21** (on the right).

Having numerous data regarding the antimicrobial activity (MICs), we determined the possible pharmacophore models that define the activity of the described compounds (Figure 5, Figure 6, Figure 7, Figure 8, Figure 9 and Figure 10).

The aldehyde **18** shows the highest activity against *Staphylococcus aureus* ATCC 43300 and the pharmacophore is defined by one hydrogen-bond acceptor (HBA, green) which is surrounded by the four excluded volumes (EV, grey) and two hydrophobes (H, cyan) comprising the chain (Figure 4). One of them is in the line designated by the hydrogen-bond acceptor at a distance 5.7 Å from it, and the other is at a distance 12.4 Å from HBA. The least active compounds (**1**, **2**, **12**, **14**, **16** and **17**), in their probable and favourable energy conformations, do not fill one hydrophobe, more distant from hydrogen-bond acceptor. Geranyl acetone (**1**) is too short. In the case of nerolidol (**2**) and its structurally related alcohols (**12**, **14**, **16** and **17**) closer to HBA, hydrophobe is filled with vinyl moiety which makes it impossible to further fill hydrophobe.

The pharmacophore model of *Enterococcus faecalis* active compounds (Figure 6) consists of one HBA, two hydrophobes and five excluded volumes. Near the hydrogen bond acceptor there should be no steric hindrance, which indicates the presence of four excluded volumes nearby. The distances from HBA to the hydrophobes are 5.8 and 7.2 Å. In this case, as in the pharmacophore model of *Staphylococcus aureus* active compounds, the least active compounds do not fill one hydrophobe, the cause are the steric and conformational factors. Furthermore, in the case of the least active ones structurally related to nerolidol **12** and **15**, it can be assumed that the short length of the molecule in conjunction with the position of the functional group which is more in the middle of the chain causes lowering of the activity of these compounds. In turn, in the case of the most active ketone **7** and acetate **35**, connection of suitable chain length and exposition of the functional group makes these compounds more active.

In the set of analysed compounds, the most potent against *Enterococcus faecium* are ketone **9** and acetate **35**. The pharmacophore comprises one hydrogen-bond acceptor, two hydrophobes and five excluded volumes (Figure 7). Both hydrophobes are in the line perpendicular to the line determined by HBA and the distances are 6.9 and 10.0 Å. The lack of filling of one hydrophobe is the cause of low activity of compounds **12** and **22**. This is due to the same considerations as in the two previous cases.

Ketone **6** shows the highest activity against the Gram-negative bacteria strains: *Escherichia coli*, *Klebsiella pneumoniae* and *Acinetobacter baumannii*. Acetate **35** shows equal activity towards the last-mentioned strain. The pharmacophores are defined by one hydrogen-bond acceptor, three (*Escherichia coli*) or two (two other strains) hydrophobes and five (*Klebsiella pneumoniae*) or two excluded volumes (Figure 8, Figure 9 and Figure 10, respectively). In the case of *E. coli*, the hydrophobes include methyl substituents at C-6 and C-7 and an isobutenyl moiety; in the case of *K. pneumoniae* the methyl groups seem to play an important role and they fit into the hydrophobes. In turn, the pharmacophore describing the spatial requirement for *A. baumannii* active molecules indicates the important role of methyl substituent at C-7 and the isobutenyl moiety. The distances from HBA to the hydrophobes are 5.5, 7.2, 9.0 and 3.4, 4.2 and 5.8, 6.1 Å (*Escherichia coli* and *Klebsiella pneumoniae* and *Acinetobacter baumannii*, respectively). In the case of pharmacophores with regard to the compounds least active against Gram-negative bacteria, the problem seems to be the position of the functional group in the chain. While both hydrophobes are filled, in each case the functional group passes the hydrogen-bond acceptor. While for compounds active against Gram-positive bacteria the exposition of the functional group is beneficial, here it does not bring the desired effect.

**Figure 11:**
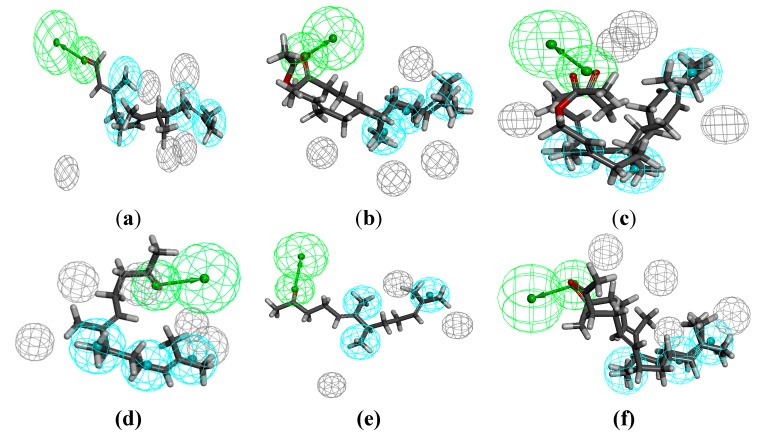
The pharmacophore models of the most active compounds against Gram-positive: *S. aureus* (**a**); *E. faecalis* (**b**); *E. faecium* (**c**) and most active compounds against Gram-negative strains: *E. coli* (**d**); *K. pneumoniae* (**e**); *A. baumannii* (**f**).

The pharmacophore modelling was conducted additionally only for the most active substances (Figure 11). The result obtained confirmed the previously described observations regarding the required length of the chain and exposition of the functional group.

Compared to the more general models obtained by using all of the compounds described herein, the above can be considered as more restrictive. Although the appearance of these models is different, their analysis leads to identical conclusions. The compounds with longer chains have higher antimicrobial activity. Additionally, exposition of the functional group is once the favourable factor (activity against Gram-positive bacteria) while it is unfavourable at another time (activity against Gram-negative bacteria).

Moreover, in the above described steric conditions, it can be seen that the β-methyl substituent also plays an important role (in the case of *S. aureus*).

### 2.3. Cytotoxicity of the Parent Terpenes and Structurally Related Compounds

All of the obtained compounds and parent terpenes as positive controls were additionally subjected to cytotoxicity (MTT test) using a popular *in vitro* model of human leukaemia HL-60 cells. We obtained the cytotoxic activities of geranyl acetone and nerolidol that were close to those reported elsewhere [11], although they possessed lower biological effectiveness. The IC_50_ level (interpolated), which means the compound concentration required to suppress cell viability by 50%, was *ca.* 135 µM and 295 µM for nerolidol (**2**) and geranyl acetone (**1**), respectively. Our studies showed that nerolidol and synthetic structurally related compounds (**12** to **17**) were more active inhibitors of cellular dehydrogenases as compared to compounds based on the geranyl acetone structure (Table 2). The IC_50_ values for the alcohols **12** to **17** were in the range of *ca.* 500 µM to 750 µM, except for **16** (*ca.* 1000 µM), while for geranyl acetone and synthetic ketones **6** to **11**, they were higher than 1000 µM.

**Table 2:** Concentration-response effects (viability) of the studied compounds on cancer and regular cells metabolic activity after 24 h.

Compound	Cancer Cell Lines			Normal Cell Lines		
	Malignant Melanoma MEWO	Colorectal Adenocarcinoma HT29	Promyelocytic Leukaemia HL‑60	Fibroblasts HFIG	Keratinocytes HaCaT	Epithelium of the Small Intestine IEC6
Geranyl acetone (**1**)	93% ± 4%	98% ± 8%	**295**	82% ± 7%	89% ± 3%	79% ± 6%
**6**	102% ± 5%	102% ± 6%	67% ± 5%	83% ± 2%	118% ± 5%	95% ± 7%
**7**	99% ± 2%	98% ± 2%	67% ± 5%	92% ± 8%	101% ± 7%	82% ± 3%
**8**	94% ± 5%	111% ± 3%	72% ± 5%	81% ± 9%	90% ± 2%	97% ± 5%
**9**	83% ± 9%	107% ± 8%	69% ± 0%	85% ± 6%	89% ± 4%	76% ± 2%
**10**	93% ± 5%	103% ± 9%	62% ± 8%	98% ± 7%	112% ± 8%	78% ± 4%
**11**	112% ± 4%	92% ± 1%	59% ± 7%	102% ± 3%	100% ± 5%	81% ± 5%
Nerolidol (**2**)	99% ± 3%	103% ± 8%	**135**	75% ± 6%	102 ± 5%	76% ± 9%
**12**	97% ± 5%	97% ± 3	**500**	110% ± 7%	101% ± 6%	73% ± 8%
**13**	83% ± 5%	99% ± 6%	**400**	87% ± 7%	87% ± 6%	96% ± 5%
**14**	99% ± 6%	112% ± 5%	**750**	99% ± 4%	106% ± 5%	65% ± 8%
**15**	104% ± 6%	104% ± 7%	**750**	96% ± 5%	90% ±7%	110% ± 3%
**16**	100% ± 7%	102% ± 3%	**950**	106% ± 5%	89% ± 4%	113% ± 2%
**17**	112% ± 0%	108% ± 4%	**750**	87% ± 5%	95% ± 0%	98% ± 5%
Farnesal (**3**)	**467**	110% ± 9%	**363**	**650**	**702**	85% ± 2%
**18**	**630**	98% ± 9%	**502**	**690**	**800**	79% ± 6%
**19**	**600**	96% ± 2%	**550**	**750**	**650**	94% ± 4%
**20**	**690**	82% ± 6%	**480**	**610**	**850**	64% ± 7%
**21**	**640**	98% ± 6%	**600**	**830**	**830**	91% ± 3%
**22**	**700**	93% ± 3%	**600**	**740**	**880**	76% ± 3%
**23**	**730**	87% ± 7%	**630**	**800**	**900**	82% ± 5%
Farnesol (**4**)	**378**	**930**	**54**	**350**	**489**	58% ± 8%
**24**	**512**	78% ± 6%	**100**	**390**	**686**	85% ± 5%
**25**	**500**	108% ± 11%	**167**	**454**	**667**	82% ± 2%
**26**	**495**	96% ± 5%	**145**	**531**	**620**	76% ± 3%
**27**	**456**	106% ± 2%	**143**	**489**	**687**	93% ± 5%
**28**	**512**	93% ± 8%	**198**	**687**	**698**	101% ± 4%
**29**	**587**	82% ± 2%	**252**	**756**	**946**	103% ± 2%
Farnesyl acetate (**5**)	**734**	105% ± 3%	**121**	98% ± 7%	102% ± 3%	78% ± 7%
**30**	**850**	96% ± 4%	**289**	105% ± 3%	**900**	86% ± 6%
**31**	**930**	107% ± 8%	**350**	**850**	98% ± 7%	94% ± 5%
**32**	**800**	103% ± 7%	**278**	**980**	**950**	92% ± 4%
**33**	103% ± 5%	110% ± 9%	**400**	100% ± 6%	101% ± 6%	95% ± 5%
**34**	98% ± 6%	98% ± 3%	**312**	99% ± 3%	110% ± 5%	79% ± 3%
**35**	112% ± 5%	83% ± 5%	**343**	87% ± 8%	102% ± 4%	71% ± 4%

The half maximal inhibitory concentrations (IC_50_) after 24 h, interpolated values (bolded). Range of the concentrations 10–1000 µM. If the IC_50_ exceeded 1000 µM viability [%] ± standard deviation at 1000 µM is given.

The very high cytotoxic activity of farnesol, and thus its potential use as an anticancer agent, was confirmed previously in studies performed on non-small cell lung cancer cells (H460 and A549), which showed that the IC_50_ values for both cell lines were approximately 4.5 µM [14]. The comparison of cell lines viability results obtained for all newly synthesised compounds revealed that farnesol and its structurally related compounds had the highest biological activity, especially against the HL-60 cell line, although none of the synthesised compound was better than farnesol (IC_50_ = 54 µM). The compounds structurally related to farnesal and farnesyl acetate were not as active as farnesol, however, they also inhibited the metabolic activity of leukaemia cells at relatively high concentrations (IC_50_ = 289 to 630 µM). All of the studied compounds were practically harmless against epithelial cell lines of digestive-system origin (colorectal adenocarcinoma HT29 and small intestine epithelium IEC6).

On the other hand, by taking into account the potential use of these new synthesised compounds in the flavour and fragrance industry, there was a need to perform cytotoxicity studies on cells of skin origin. It is known that the skin consists of different types of cells, such as keratinocytes, melanocytes and fibroblasts; thus the compounds’ influence on cellular metabolic activity was measured on MEWO melanocytes, immortalised keratinocytes HaCaT and normal human fibroblast cell lines (HFIG). In this case, the IC_50_ levels against the cell lines used could not be obtained for geranyl acetone, nerolidol and their structurally related compounds within the range of the tested compound concentrations; thus these values were higher than 1000 µM. What is more, the maximum metabolic inhibitory effect observed for the highest compound concentration did not exceed 15% (data not shown). Studies performed by Pohlit *et al.* indicated no toxicity of nerolidol against other human melanoma MDA/MB‑435 cell lines and colon carcinoma HCT-8 cells [12]. These data suggest that the obtained compounds could probably be safely used in flavour and fragrance compositions. The compounds structurally related to farnesol, farnesal and farnesyl acetate influenced skin cells viability and their IC_50_ values were in the range of 350 to 990 µM; the keratinocytes were the least sensitive from the studied cell lines, however, the results obtained for normal fibroblasts and malignant cells were comparable.

When comparing the results it was important to emphasise that the HL-60 cell line was more sensitive to the tested compounds, whereas skin and digestive-system originated cells (both normal and immortalised) did not show a significant reduction of metabolic activity. Using HL-60 cells as a single cellular model may give a false evaluation of the biological activity of these compounds.

## 3. Experimental Section

### 3.1. General

The reagents were generally of commercial grade and were used without further purification. Column chromatography on silica gel for TLC with hexane: ethyl acetate (9:1) as the mobile phase (unless otherwise indicated). GC-MS (Pegasus 4D, LECO, St. Joseph, MI, USA) was performed with a chromatograph coupled with an MS detector, equipped with a BPX-5 (SGE Analytical Science, Ringwood, Melbourne, Vic, Australia) capillary column (30 m long, 0.25 mm inside diameter, 0.25 µm film thickness); the temperature programme was 50 to 300 °C at 4°/min, carrier gas: helium, flow 1 mL/min. ^1^H-NMR (250 MHz) and ^13^C-NMR (63 MHz) of the fractions enriched with the individual *E*/*Z* isomers and racemates in the case of **23**, **29** and **35** were recorded using a Bruker DPX-250 Avance spectrometer in CDCl_3_ solutions with TMS as the internal standard. Some NMR spectra were recorded using a Bruker Advance II Plus 700 MHz (BRUKER, Billerica, MA, USA). ^13^C-NMR multiplicity was determined using DEPT experiments. Purity of the products was confirmed by both GC and TLC analyses. The samples on a blotter (10% ethanolic solutions) were judged by nine experienced panellists to evaluate the odour characteristics of the obtained products.

#### 3.1.1. Antimicrobial Test

The standard strains, Gram-positive: *Staphylococcus aureus* ATCC 433000, *Enterococcus faecalis* Van B ATCC 51299 and *Enterococcus faecium* VA sensitive ATCC 35667, and Gram-negative: *Escherichia coli* ATCC 25922, *Klebsiella pneumoniae* ATCC 700603 and *Acinetobacter baumannii* ATCC 19606, came from a collection of the Environmental Biology Department, Medical University of Łódź. The standard strains were cultivated in Columbia Agar Medium and incubated at 37 °C for 24 h in aerobic conditions.

The antibacterial activity of the racemates and *E*/*Z*-mixtures was investigated using the micro‑dilution broth method [23]. The compounds were diluted in ethanol. This stock solution was mixed with 100 µL of Mueller-Hinton broth to obtain concentrations from 10 µg/mL to 80 µg/mL. The mixtures were then transferred to 96-well microtiter plates. The microbial suspension was standardised to a cell density of 1.5 × 10^8^ CFU, which was equal to an optical density of 0.5 on the Mc Farland scale by a bio-Merieux densitometer; one well was filled with broth only to act as a control for strain growth. The Minimal Inhibitory Concentration (MIC) was determined as the lowest concentration of the compound which inhibited visible growth of bacteria after 24 h of incubation at 37 °C under aerobic conditions. The control media containing only alcohol at concentrations used in the dilutions of the compounds did not inhibit the growth of the standard.

#### 3.1.2. *In Vitro* Inhibition of Animal Cell Line Viability

*Cell lines and in vitro culture conditions:* The human promyelocytic leukaemia cell line HL-60 was obtained from the Centre of Molecular and Macromolecular Biology in Łódź, Poland and was cultured in RPMI 1640 medium with 20% heat-inactivated fetal bovine serum (FBS). To reduce spontaneous differentiation, the cells were never allowed to exceed a concentration of 1.0 × 10^6^ cells/mL. The human keratinocyte cell line HaCaT was obtained from Cell Lines Service, Germany (CLS) and cultured in DMEM medium with 10% FBS. Human colorectal adenocarcinoma HT29 and normal epithelial IEC-6 cell lines (from ATCC) were cultured in DMEM medium with 10% FBS. The human skin melanoma cell line MEWO (from CLS) was cultured in DMEM:F12 supplemented with 2 mM l-glutamine and 10% FBS. Human fibroblasts from gingiva isolated from original tissue, HFIB-G, (from Provitro) were cultured in Quantum 333 (from Immunique). The media were supplemented with 100 U/mL penicillin and 100 µg/mL streptomycin; all media components were obtained from Life Technologies. The adherent cell lines used in the studies were seeded on microplates before they exceeded 80% of confluence. Cells were cultured at 37 °C in 5% CO_2_ air atmosphere/95% air atmosphere.

*Cytotoxic activity*: The cells (the density for HL-60 was 0.5 × 10^4^ cells/100 µL, otherwise it was 1 × 10^4^ cells/100 µL) were plated into each well of 96-well microtiter plates and, after their attachment to the plate surface, treated with 10 µL of the indicated concentrations of compounds for 24 h. Viability was determined by MTT assay following the treatment. MTT (3-[4.5-dimethylthiazol-2-yl]-2.5-diphenyltetrazolium bromide; Sigma, St. Louis, MO, USA) solution was added to each well and incubated for another 4 h. The resulting MTT-formazan product was dissolved by the addition of lysis buffer (20% SDS and 50% DMF). The amount of formazan was determined by measuring absorbance at 570 nm with a Synergy 2 microplate reader (BioTek, Winooski, VT, USA) operated by the Gen5 program. The experiments were conducted three times with five replications. The results are expressed as an optical density ratio of the treatment to control. All data are presented as means ± SD.

#### 3.1.3. Pharmacophore Modelling

Optimised models of all possible isomers of described compounds (including parent terpenes) with the density functional theory (DFT) and *ab initio* molecular dynamics calculation at B3LYP/6-31G(d) (Gaussian 03W v. 6.1) were used as ligands in 3D QSAR pharmacophore modelling which was performed using Discovery Studio 3.0 Client software (Polish National Licence, BIOVIA, San Diego, CA, USA). MICs were used as the activity attribute. The value of uncertainty was set at 1.5. The pharmacophore generation protocol settings were as follows (as recommended in the Discovery Studio 3D QSAR Pharmacophore Generation Manual and due to the modelling of relatively small molecules): the number of features (hydrogen bond acceptor, hydrogen bond donor, hydrophobic, positive and negative ionizable) from 0 to 5; maximum pharmacophores 10; minimum features 1; maximum features 5; minimum interfeature distance 1.5; maximum excluded volumes 5; minimum feature points 4; minimum subset points 4; conformation generation BEST; maximum conformations 255. From the resulting pool of generated pharmacophores, the one that characterized the highest correlation was chosen.

### 3.2. General Method for Rearrangement of the Tertiary Allylic Alcohols Combined with Oxidation to Aldehydes-Synthesis of Compounds Structurally Related to Farnesal (***18***–***23***)

A total of 340 mmol of the appropriate allylic alcohol (**12**–**17**), 150 mL of dichloromethane and 50 g of silica gel was placed into a flask. During vigorous stirring, 410 mmol of pyridinium chlorochromate (PCC) was added portion-wise. The temperature was maintained at 10 °C. After addition, the stirring was continued for another 12 h. After that time, 10 mL of methanol was added and the mixture was stirred again for 1 h. The products were isolated by column chromatography with diethyl ether as eluent. Purity of the products according to the GC analysis was 88%–97%. The obtained products (**18**–**23**) were additionally distilled or purified by column chromatography (see below).

#### 3.2.1. 3,7,8,12-Tetramethyltrideca-2,6,11-trienal (**18**)

Fractional distillation (boiling point 131–134 °C/0.1 Torr) gave the product with a yield 43%. When the product was isolated by column chromatography on silica (hexane:ethyl acetate 96:4) the yield was 72%. IR (ATR): 2961, 2922, 2856, 2765, 1674, 1633, 1445, 1377, 1156, 1121, 830 cm^−1^; ^1^H-NMR (250 MHz, CDCl_3_) Isomer (2*Z*,6*Z*) δ: 9.90 (d, *J* = 8.2 Hz, 1H), 5.85 (dd, *J* = 8.2 Hz, 1.2 Hz, 1H), 5.14–5.00 (m, 2H), 2.56 (dd, *J* = 15.1, 7.5 Hz, 2H), 2.57–2.45 (m, 1H), 2.28–2.16 (m, 2H), 1.95 (d, *J =* 1.2 Hz, 3H), 1.80 (dd, *J =* 14.6 Hz, 7.1 Hz, 2H), 1.64 (s, 3H), 1.54 (s, 3H), 1.53 (s, 3H), 1.37–1.20 (m, 2H), 0.92 (d, *J =* 6.9 Hz, 3H); Isomer (2*Z*,6*E*) δ: 9.88 (d, *J =* 8.2 Hz, 1H), 5.85 (dd, *J =* 8.2 Hz, 1.2 Hz, 1H), 5.14–5.00 (m, 2H), 2.57 (t, *J =* 7.5 Hz, 3H), 2.28–2.16 (m, 2H), 2.05 (dd, *J =* 14.4 Hz, 6.9 Hz, 1H), 1.96 (d, *J =* 1.2 Hz, 3H), 1.80 (dd, *J =* 14.6 Hz, 7.1 Hz, 2H), 1.64 (s, 3H), 1.54 (s, 3H), 1.48 (s, 3H), 1.37–1.20 (m, 2H), 0.92 (d, *J =* 6.9 Hz, 3H); Isomer (2*E*,6*Z*) δ: 9.95 (d, *J =* 8.1 Hz, 1H), 5.85 (dd, *J =* 8.1 Hz, 1.1 Hz, 1H), 5.10–5.00 (m, 2H), 2.62–2.48 (m, 1H), 2.27–2.15 (m, 4H), 2.14 (d, *J =* 1.1 Hz, 3H), 1.81 (dd, *J =* 14.9 Hz, 7.1 Hz, 2H), 1.64 (s, 3H), 1.54 (s, 3H), 1.53 (s, 3H), 1.39–1.16 (m, 2H), 0.92 (d, *J =* 6.9 Hz, 3H); Isomer (2*E*,6*E*) δ: 9.95 (d, *J =* 8.1 Hz, 1H), 5.85 (dd, *J =* 8.1 Hz, 1.1 Hz, 1H), 5.10–5.00 (m, 2H), 2.28–2.15 (m, 4H), 2.14 (d, *J =* 1.1 Hz, 3H), 2.05 (dd, *J =* 14.5 Hz, 6.9 Hz, 1H), 1.81 (dd, *J =* 14.9 Hz, 7.1 Hz, 2H), 1.64 (s, 3H), 1.54 (s, 3H), 1.48 (s, 3H), 1.39–1.16 (m, 2H), 0.92 (d, *J =* 6.9 Hz, 3H); ^13^C-NMR (63 MHz, CDCl_3_) Isomer (2*Z*,6*Z*) δ: 190.5 (d), 163.6 (s), 141.0 (s), 131.2 (s), 128.5 (d), 124.5 (d), 122.8 (d), 34.7 (t), 33.4 (d), 32.8 (t), 26.2 (t), 26.1 (t), 25.5 (q), 24.9 (q), 19.1 (q), 17.8 (q), 17.5 (q); Isomer (2*Z*,6*E*) δ: 190.6 (d), 163.8 (s), 141.2 (s), 130.9 (s), 128.5 (d), 124.6 (d), 121.5 (d), 42.2 (d), 34.9 (t), 32.4 (t), 26.7 (t), 26.0 (t), 25.5 (q), 25.3 (q), 19.5 (q), 17.5 (q), 12.2 (q); Isomer (2*E*,6*Z*) δ: 191.0 (d), 163.6 (s), 140.4 (s), 131.1 (s), 127.3 (d), 124.5 (d), 123.0 (d), 40.8 (t), 34.7 (t), 33.4 (d), 26.1 (t), 25.5 (q), 24.9 (t), 19.1 (q), 17.8 (q), 17.5 (q), 17.4 (q); Isomer (2*E*,6*E*) δ: 191.0 (d), 163.6 (s), 140.5 (s), 130.9 (s), 127.3 (d), 124.6 (d), 121.9 (d), 42.2 (d), 40.5 (t), 34.9 (t), 26.0 (t), 25.5 (q), 25.3 (t), 19.6 (q), 17.5 (q), 17.4 (q), 12.1 (q); MS (70 eV) *m/z* (%) Isomer (2*Z*,6*Z*) 248 [M]^+^ (1), 82 (100), 69 (53), 95 (45), 41 (38), 109 (38), 55 (36), 67 (33), 81 (25), 83 (25), 149 (12), 121 (11), 137 (10), 167 (5), 205 (4); Isomer (2*Z*,6*E*) 248 [M]^+^ (1), 82 (100), 69 (45), 95 (30), 41 (29), 109 (29), 55 (28), 67 (27), 83 (21), 81 (20), 149 (8), 121 (7), 137 (6), 167 (5), 205 (2); Isomer (2*E*,6*Z*) 248 [M]^+^ (1), 82 (100), 69 (53), 95 (35), 41 (33), 109 (30), 67 (30), 55 (29), 81 (22), 83 (22) 149 (10), 121 (8), 137 (7), 167 (5), 205 (2); Isomer (2*E*,6*E*) 248 [M]^+^ (1), 82 (100), 69 (40), 41 (26), 67 (26), 95 (25), 55 (24), 109 (23), 83 (19), 81 (17), 167 (7), 149 (6), 121 (5), 205 (1). Anal. Calcd for: C_17_H_28_O: C, 82.20; H, 11.36. Found: C, 82.48; H, 11.32.

#### 3.2.2. (6*E*)-3,8,12-Trimethyltrideca-2,6,11-trienal (**19**)

Column chromatography on silica gel (hexane:ethyl acetate 98:2) gave a yield of 71%. IR (ATR) 2965, 2915, 2853, 2764, 1673, 1633, 1449, 1377, 1193, 1123, 970, 841 cm^−1^; ^1^H-NMR (250 MHz, CDCl_3_) Isomer (2*Z*,6*E*) δ: 9.92 (d, *J =* 8.2 Hz, 1H), 5.88 (dd, *J =* 8.2 Hz, 1.2 Hz, 1H), 5.36–5.29 (m, 2H), 5.08 (dqq, *J =* 7.1, 1.1, 1.1 Hz, 1H), 2.63 (t, *J =* 7.5 Hz, 2H), 2.30–2.19 (m, 2H), 2.11–2.01 (m, 1H), 1.97 (d, *J =* 1.2 Hz, 3H), 1.96–1.86 (m, 2H), 1.67 (d, *J =* 1.1 Hz, 3H), 1.58 (s, 3H), 1.26 (dd, *J =* 15.3 Hz, 7.1 Hz, 2H), 0.94 (d, *J =* 6.7 Hz, 3H); Isomer (2*E*,6*E*) δ: 9.98 (d, *J =* 8.1 Hz, 1H), 5.87 (dd, *J =* 8.1 Hz, 1.2 Hz, 1H), 5.35-5.28 (m, 2H), 5.07 (dqq, *J =* 7.1, 1.0, 1.0 Hz, 1H), 2.32–2.18 (m, 4H), 2.15 (d, *J =* 1.2 Hz, 3H), 2.10–2.00 (m, 1H), 1.95–1.84 (m, 2H), 1.67 (d, *J =* 1.0 Hz, 3H), 1.57 (s, 3H), 1.25 (dd, *J =* 15.4 Hz, 7.1 Hz, 2H), 0.93 (d, *J =* 6.8 Hz, 3H); ^13^C-NMR (63 MHz, CDCl_3_) Isomer (2*Z*,6*E*) δ: 190.3 (d), 163.1 (s), 138.1 (d), 130.7 (s), 128.5 (d), 125.9 (d), 124.4 (d), 36.8 (t), 35.9 (d), 32.5 (t), 31.3 (t), 25.5 (t), 25.4 (q), 20.7 (q), 20.4 (q), 17.4 (q); Isomer (2*E*,6*E*) δ: 190.7 (d), 162.9 (s), 137.5 (d), 130.7 (s), 127.3 (d), 126.3 (d), 124.4 (d), 40.4 (t), 36.8 (t), 36.0 (d), 29.9 (t), 25.5 (t), 25.4 (q), 20.5 (q), 17.4 (q), 17.2 (q); MS (70 eV) *m*/*z* (%) Isomer (2*Z*,6*E*) 234 [M]^+^ (2), 69 (100), 82 (82), 41 (81), 81 (77), 95 (69), 84 (61), 55 (57), 67 (53), 107 (34), 109 (32), 135 (22), 123 (21), 121 (20), 150 (16), 219 (6); Isomer (2*E*,6*E*) 234 [M]^+^ (1), 69 (100), 84 (96), 81 (83), 82 (71), 41 (71), 95 (60), 55 (59), 67 (53), 107 (29), 109 (28), 150 (25), 151 (24), 135 (17), 121 (13). Anal. Calcd for: C_16_H_26_O: C, 81.99; H, 11.18. Found: C, 81.69; H, 11.15.

#### 3.2.3. 3,7,8,12-Tetramethyltrideca-2,6-dienal (**20**)

Boiling point 120–124 °C/0.1 Torr. Yield 36%. Isolation by column chromatography gave a product with a yield of 83%. IR (ATR) 2955, 2926, 2868, 2775, 1675, 1456, 1381, 1192, 1121, 852 cm^−1^; ^1^H-NMR (250 MHz, CDCl_3_) Isomer (2*Z*,6*Z*) δ: 9.91 (d, *J =* 8.3 Hz, 1H), 5.87 (d, *J =* 8.3 Hz, 1H), 5.15–5.05 (m, 1H), 2.77–2.64 (m, 2H), 2.59 (t, *J =* 7.4 Hz, 2H), 2.30–2.17 (m, 2H), 1.97 (s, 3H), 1.57–1.40 (m, 1H), 1.54 (s, 3H), 1.30–1.06 (m, 6H), 0.93 (d, *J =* 6.9 Hz, 3H), 0.86–0.81 (m, 6H); Isomer (2*Z*,6*E*) δ: 9.92 (d, *J =* 8.3 Hz, 1H), 5.87 (d, *J =* 8.3 Hz, 1H), 5.15–5.05 (m, 1H), 2.59 (t, *J =* 7.4 Hz, 2H), 2.30–2.17 (m, 2H), 2.10–1.92 (m, 2H), 1.97 (s, 3H), 1.57–1.40 (m, 1H), 1.50 (s, 3H), 1.30–1.06 (m, 6H), 0.93 (d, *J =* 6.9 Hz, 3H), 0.86–0.81 (m, 6H); Isomer (2*E*,6*Z*) δ: 9.98 (d, *J =* 8.1 Hz, 1H), 5.85 (d, *J =* 8.1 Hz, 1H), 5.11–4.99 (m, 1H), 2.77–2.64 (m, 2H), 2.30–2.17 (m, 3H), 1.97 (s, 3H), 1.57–1.40 (m, 1H), 1.54 (s, 3H), 1.30–1.06 (m, 6H), 0.93 (d, *J =* 6.9 Hz, 3H), 0.86–0.81 (m, 6H); Isomer (2*E*,6*E*) δ: 9.98 (d, *J =* 8.1 Hz, 1H), 5.85 (d, *J =* 8.1 Hz, 1H), 5.11–4.99 (m, 1H), 2.30–2.17 (m, 3H), 2.10–1.92 (m, 2H), 1.97 (s, 3H), 1.57–1.40 (m, 1H), 1.50 (s, 3H), 1.30–1.06 (m, 6H), 0.93 (d, *J =* 6.9 Hz, 3H), 0.86–0.81 (m, 6H); ^13^C-NMR (63 MHz, CDCl_3_) Isomer (2*Z*,6*Z*) δ: 190.6 (d), 163.7 (s), 141.3 (s), 128.5 (d), 122.5 (d), 39.1 (t), 34.9 (t), 33.9 (d), 32.9 (t), 27.8 (d), 26.3 (t), 25.4 (t), 24.9 (q), 22.6 (q), 22.5 (q), 19.2 (q), 17.9 (q); Isomer (2*Z*,6*E*) δ: 190.7 (d), 163.9 (s), 141.5 (s), 128.5 (d), 121.2 (d), 42.6 (d), 39.0 (t), 35.0 (t), 32.5 (t), 27.8 (d), 26.7 (t), 25.2 (t), 24.9 (q), 22.6 (q), 22.5 (q), 19.5 (q), 12.5 (q); Isomer (2*E*,6*Z*) δ: 191.1 (d), 163.7 (s), 140.7 (s), 127.3 (d), 122.8 (d), 40.9 (t), 39.1 (t), 34.8 (t), 33.9 (d), 27.8 (d), 25.4 (t), 25.0 (t), 22.6 (q), 22.5 (q), 19.2 (q), 17.9 (q), 17.4 (q); Isomer (2*E*,6*E*) δ: 191.1 (d), 163.8 (s), 140.8 (s), 127.3 (d), 122.6 (d), 42.6 (d), 40.5 (t), 38.9 (t), 35.0 (t), 27.8 (d), 25.3 (t), 25.2 (t), 22.6 (q), 22.5 (q), 19.7 (q), 17.4 (q), 12.2 (q); MS (70 eV) *m*/*z* (%) Isomer (2*Z*,6*Z*) 250 [M]^+^ (1), 69 (100), 55 (87), 95 (70), 109 (58), 81 (47), 82 (47), 83 (47), 41 (43), 137 (43), 43 (41), 123 (20), 157 (12), 166 (11), 147 (11), 235 (3); Isomer (2*Z*,6*E*) 250 [M]^+^ (4), 69 (100), 55 (62), 111 (36), 83 (36), 81 (35), 97 (35), 41 (28), 43 (25), 165 (17), 137 (15), 221 (4); Isomer (2*E*,6*Z*) 250 [M]^+^ (1), 69 (100), 55 (84), 95 (64), 109 (51), 81 (46), 83 (46), 137 (39), 41 (39), 43 (37), 123 (15), 157 (12), 165 (11), 147 (10), 235 (3); Isomer (2*E*,6*E*) 250 [M]^+^ (2), 69 (100), 55 (70), 111 (38), 97 (34), 83 (34), 81 (30), 57 (30), 41 (28), 43 (25), 137 (14), 165 (11), 221 (3). Anal. Calcd for: C_17_H_30_O: C, 81.54; H, 12.07. Found: C, 81.96; H, 12.15.

#### 3.2.4. 3,5,9-Trimethyldeca-2,8-dienal (**21**)

B.P. 90–92 °C/0.17 Torr. Yield 54%. IR (ATR) 2964, 2918, 2853, 2767, 1671, 1629, 1449, 1378, 1195, 1122, 867 cm^−1^; ^1^H-NMR (250 MHz, CDCl_3_) Isomer *Z* δ: 9.91 (d, *J =* 8.3 Hz, 1H), 5.83 (dd, *J =* 8.3 Hz, 1.2 Hz, 1H), 5.05 (dqq, *J =* 7.1, 1.3, 1.3 Hz, 1H), 2.45 (d, *J =* 7.4 Hz, 2H), 2.01 (dd, *J =* 16.0 Hz, 7.1 Hz, 3H), 1.92 (d, *J =* 1.2 Hz, 3H), 1.73 (dd, *J =* 13.3 Hz, 6.8 Hz, 1H), 1.66 (d, *J =* 1.3 Hz, 3H), 1.58 (s, 3H), 1.43–1.12 (m, 2H), 0.89 (d, *J =* 6.8 Hz, 3H); Isomer *E* δ: 9.96 (d, *J =* 8.1 Hz, 1H), 5.83 (dd, *J =* 8.1 Hz, 1.2 Hz, 1H), 5.05 (dqq, *J =* 7.1, 1.3, 1.3 Hz, 1H), 2.21 (dd, *J =* 13.3 Hz, 6.1 Hz, 1H), 2.10 (d, *J =* 1.2 Hz, 3H), 2.02–1.90 (m, 3H), 1.72 (dd, *J =* 13.4 Hz, 6.7 Hz, 1H), 1.66 (d, *J =* 1.3 Hz, 3H), 1.58 (s, 3H), 1.40–1.05 (m, 2H), 0.84 (d, *J =* 6.7 Hz, 3H); ^13^C-NMR (63 MHz, CDCl_3_) Isomer *Z* δ: 190.8 (d), 163.5 (s), 131.6 (s), 129.7 (d), 124.0 (d), 39.8 (t), 36.9 (t), 31.5 (d), 25.6 (q), 25.4 (t), 25.1 (q), 19.3 (q), 17.6 (q); Isomer *E* δ: 191.1 (d), 163.2 (s), 131.5 (s), 128.6 (d), 124.1 (d), 48.5 (t), 36.8 (t), 30.5 (d), 25.6 (q), 25.3 (t), 19.3 (q), 17.6 (q), 17.3 (q); MS (70 eV) *m*/*z* (%) Isomer *Z* 194 [M]^+^ (3), 109 (100), 95 (88), 69 (87), 41 (83), 55 (56), 111 (49), 151 (43), 67 (37), 81 (35), 84 (35), 43 (31), 123 (28), 138 (25), 179 (18); Isomer *E* 194 [M]^+^ (3), 69 (100), 41 (77), 95 (69), 109 (67), 55 (53), 111 (50), 67 (32), 84 (30), 179 (25), 81 (25), 107 (24), 123 (23), 137 (22), 151 (16). Anal. Calcd for: C_13_H_22_O: C, 80.35; H, 11.41. Found: C, 79.96; H, 11.49.

#### 3.2.5. 3,6,10-Trimethylundeca-2,9-dienal (**22**)

B.P. 90–94 °C/0.04 Torr. Yield 46%. IR (ATR) 2961, 2917, 2855, 2764, 1673, 1633, 1448, 1378, 1129, 839 cm^−1^; ^1^H-NMR (250 MHz, CDCl_3_) Isomer *Z* δ: 9.96 (d, *J* = 8.3 Hz, 1H), 5.83 (dd, *J =* 8.3 Hz, 1.2 Hz, 1H), 5.08 (t, *J* = 7.1 Hz, 1H), 2.56 (t, *J* = 7.4 Hz, 2H), 2.02-1.92 (m, 2H), 1.97 (d, *J* = 1.2 Hz, 3H), 1.68 (s, 3H), 1.60 (s, 3H), 1.50–1.42 (m, 1H), 1.40–1.32 (m, 2H), 1.20–1.13 (m, 2H), 0.93 (d, *J* = 6.6 Hz, 3H); Isomer *E* δ: 9.98 (d, *J* = 8.1 Hz, 1H), 5.85 (dd, *J =* 8.1 Hz, 1.1 Hz, 1H), 5.08 (t, *J* = 7.1 Hz, 1H), 2.22–2.16 (m, 2H), 2.10 (d, *J* = 1.1 Hz, 3H), 1.98 (dd, *J =* 14.5 Hz, 7.1 Hz, 2H), 1.68 (s, 3H), 1.60 (s, 3H), 1.50–1.42 (m, 1H), 1.40–1.32 (m, 2H), 1.20–1.13 (m, 2H), 0.95 (d, *J* = 6.5 Hz, 3H); ^13^C-NMR (63 MHz, CDCl_3_) Isomer *Z* δ: 190.8 (d), 165.7 (s), 131.3 (s), 127.9 (d), 124.4 (d), 36.6 (t), 36.2 (t) 32.4 (d), 30.2 (t), 25.6 (q), 25.3 (t), 25.0 (q), 19.3 (q), 17,5 (q); Isomer *E* δ: 190.7 (d), 165.3 (s), 130.9 (s), 127.9 (d), 124.8 (d), 36.7 (t), 36.3 (t), 36.0 (t), 32.7 (d), 25.6 (q), 25.4 (t), 19.2 (q), 17.5 (q), 17.5 (q); MS (70 eV) *m*/*z* (%) Isomer *Z*: 208 [M]^+^ (4), 69 (100), 97 (90), 41 (81), 84 (67), 109 (64), 95 (57), 55 (47), 82 (35), 67 (34), 123 (27), 193 (21); Isomer *E*: 208 [M]^+^ (8), 69 (100), 97 (97), 41 (77), 84 (52), 55 (49), 109 (48), 95 (44), 67 (30), 81 (28), 123 (26), 193 (13). Anal. Calcd for: C_14_H_24_O: C, 80.71; H, 11.61. Found: C, 80.62; H, 11.71.

#### 3.2.6. 3,4,6,10-Tetramethylundeca-2,9-dienal (**23**)

Boiling point 115–117 °C/0.16 Torr. Yield 67%. IR (ATR) 2964, 2916, 2873, 2851, 2756, 1672, 1632, 1455, 1378, 1198, 1135, 858 cm^−1^; ^1^H-NMR (250 MHz, CDCl_3_) *Z* Isomers δ: 10.06 (d, *J =* 8.3 Hz, 1H), 5.83 (d, *J =* 8.3 Hz, 1H), 5.13–5.00 (m, 1H), 3.63–3.50 (m, 1H), 2.04–1.89 (m, 2H), Racemate 1 and 2: 1.87 and 1.85 (d, *J =* 1.2 Hz, 3H), 1.67 (s, 3H), 1.59 (s, 3H), 1.55–1.22 (m, 5H), Racemate 1 and 2: 1.12 and 1.07 (d, *J =* 6.7 Hz, 3H), 0.91–0.86 (m, 3H); *E* Isomers δ: 10.01 (d, *J =* 8.0 Hz, 1H), 5.89 (dd, *J =* 8.0 Hz, 1.2 Hz, 1H), 5.06 (dqq, *J =* 7.0, 1.2, 1.2 Hz, 1H), 2.38 (m, 1H), Raemate 4: 2.12 (d, *J =* 1.2 Hz, 3H), Racemate 3: 2.09 (d, *J =* 1.2 Hz, 3H), 2.03–1.90 (m, 2H), 1.67 (d, *J =* 1.2 Hz, 3H), 1.59 (d, *J =* 1.2 Hz, 3H), 1.52–1.15 (m, 5H), Racemate 3: 1.07 (d, *J =* 6.8 Hz, 3H), Racemate 4: 1.85 (d, *J =* 6.8 Hz, 3H), 0.87 (d, *J =* 6.3 Hz, 3H); ^13^C-NMR (63 MHz, CDCl_3_) *Z* Racemate 1 and 2 δ: 189.9 (d), 189.8 (d), 168.9 (s), 168.4 (s), 131.3 (s), 131.2 (s), 129.1 (d), 128.4 (d), 124.6 (d), 124.4 (d), 42.3 (t), 41.8 (t), 37.5 (t), 36.7 (t), 32.3 (d), 30.2 (d), 30.2 (d), 25.6 (q), 25.3 (t), 19.8 (q), 19.4 (q), 19.2 (q), 19.0 (q), 18.6 (q), 18.6 (q), 17.6 (q); *E* Racemate 3 δ: 191.6 (d), 168.5 (s), 131.3 (s), 127.0 (d), 124.5 (d), 41.9 (t), 41.2 (d), 37.3 (t), 30.1 (d), 25.6 (q), 25.3 (t), 19.7 (q), 19.5 (q), 17.6 (q), 13.8 (q); *E* Racemate 4 δ: 191.6 (d), 169.1 (s), 131.3 (s), 126.5 (d), 124.5 (d), 42.2 (t), 41.1 (d), 36.9 (t), 30.0 (d), 25.6 (q), 25.2 (t), 19.6 (q), 18.7 (q), 17.6 (q), 14.6 (q); MS (70 eV) *m*/*z* (%) Racemate 1 (*Z*) 222 [M]^+^ (2), 69 (100), 41 (77), 109 (70), 111 (60), 55 (47), 95 (44), 85 (41), 83 (40), 98 (36), 43 (33), 149 (24), 152 (30), 123 (19), 166 (10), 207 (6); Racemate 2 (*Z*) 222 [M]^+^ (3), 69 (100), 41 (87), 111 (77), 109 (70), 55 (50), 98 (47), 95 (47), 83 (41), 152 (40), 43 (33), 67 (32), 149 (26), 123 (19), 166 (12), 207 (7); Racemate 3 (*E*) 222 [M]^+^ (3), 69 (100), 111 (91), 41 (77), 109 (60), 98 (43), 55 (42), 95 (37), 83 (32), 152 (29), 81 (28), 67 (27), 43 (21), 149 (19), 123 (14), 207 (6); Racemate 4 (*E*) 222 [M]^+^ (4), 69 (100), 111 (78), 41 (77), 109 (60), 98 (39), 55 (39), 95 (34), 83 (30), 81 (27), 67 (26), 152 (24), 43 (21), 149 (20), 123 (14), 166 (6). Anal. Calcd for: C_15_H_26_O: C, 81.02; H, 11.79. Found: C, 80.94; H, 11.65.

### 3.3. Sodium Borohydride Reduction of Aldehydes Structurally Related to Farnesol *(**24**–**29**)*

A total of 0.11 g (3 mmol) of sodium borohydride was added portion-wise to the stirred solution of 5.5 mmol of appropriate aldehyde (**18**–**23**) dissolved in 10 mL of isopropanol. After addition, the reaction mixture was stirred for 24 h. Next isopropanol was removed by evaporation and the residue was transferred to the separatory funnel, after which 20 mL of water and 30 mL of hexane was added. After separation of the organic layer, the water layer was extracted with hexane (2 × 20 mL). The combined extracts were washed with brine and dried over anhydrous magnesium sulphate. The product was purified by column chromatography on silica gel using hexane:ethyl acetate (97:3), or distilled (see below).

#### 3.3.1. 3,7,8,12-Tetramethyltrideca-2,6,11-trien-1-ol (**24**)

Yield 87%. IR (ATR) 3306, 2962, 2919, 2856, 1667, 1448, 1376, 1001, 829 cm^−1^. ^1^H-NMR (250 MHz, CDCl_3_) Isomer (2*Z*,6*Z*) and (2*Z*,6*E*) δ: 5.58–5.39 (m, 1H), 5.15–5.00 (m, 2H), 4.11 (d, *J =* 7.1 Hz, 2H), 2.18–1.98 (m, 5H), 1.85 (dd, *J =* 14.7 Hz, 7.2 Hz, 2H), 1.75 (d, *J =* 1.3 Hz, 3H), 1.67 (d, *J =* 0.9 Hz, 3H), 1.58 (s, 3H), 1.51 (d, *J =* 1.0 Hz, 3H), 1.40–1.15 (m, 2H), 0.96 (d, *J =* 6.9 Hz, 3H); Isomer (2*E*,6*Z*) and (2*E*,6*E*) δ: 5.45–5.37 (m, 1H), 5.15–5.05 (m, 2H), 4.14 (d, *J =* 6.9 Hz, 2H), 2.16–2.00 (m, 5H), 1.85 (dd, *J =* 14.7 Hz, 7.1 Hz, 2H), 1.68 (s, 6H), 1.58 (s, 3H), 1.51 (d, *J =* 0.9 Hz, 3H), 1.42–1.21 (m, 2H), 0.96 (d, *J =* 6.9 Hz, 3H); ^13^C-NMR (63 MHz, CDCl_3_) Isomer (2*Z*, 6*Z*) δ: 140.0 (s), 139.6 (s), 131.2 (s), 124.7 (d), 124.4 (d), 124.4 (d), 58.9 (t), 34.9 (t), 33.5 (d), 32.3 (t), 26.2 (t), 25.7 (t), 25.6 (q), 23.4 (q), 19.2 (q), 17.9 (q), 17.6 (q); Isomer (2*Z*,6*E*) δ: 139.8 (s), 139.8 (s), 131.0 (s), 124.8 (d), 124.3 (d), 123.0 (d), 58.9 (t), 42.3 (d), 35.0 (t), 32.0 (t), 26.3 (t), 26.1 (t), 25.6 (q), 23.4 (q), 19.6 (q), 17.6 (q), 12.2 (q); Isomer (2*E*,6*Z*) δ: 139.6 (s), 139.4 (s), 131.1 (s), 124.8 (d), 124.4 (d), 123.4 (d), 59.2 (t), 39.9 (t), 34.9 (t), 33.5 (d), 26.2 (t), 25.7 (t), 25.6 (q), 19.2 (q), 17.9 (q), 17.6 (q), 16.2 (q); Isomer (2*E*,6*E*) δ: 139.6 (s), 139.4 (s), 131.0 (s), 124.8 (d), 123.4 (d), 123.3 (d), 59.2 (t), 42.3 (d), 39.5 (t), 35.0 (t), 26.1 (t), 26.0 (t), 25.6 (q), 19.7 (q), 17.6 (q), 16.2 (q), 12.2 (q); MS (70 eV) *m*/*z* (%) 250 [M]^+^ (1), 232 ([M]^+^-18, 2), 82 (100), 69 (69), 109 (40), 95 (33), 81 (27), 41 (26), 55 (25), 83 (25), 121 (16), 168 (4), 217 (2). Anal. Calcd for: C_17_H_30_O: C, 81.54; H, 12.07. Found: C, 81.46; H, 12.02.

#### 3.3.2. (6*E*)-3,8,12-Trimethyltrideca-2,6,11-trien-1-ol (**25**)

Yield 74%. IR (ATR): 3340, 2965, 2916, 2855, 1670, 1450, 1377, 997, 968, 911, 733 cm^−1^. ^1^H-NMR (250 MHz, CDCl_3_) Isomer (2*Z*,6*E*) δ: 5.47–5.38 (m, 1H), 5.35–5.20 (m, 2H), 5.12–5.04 (m, 1H), 4.10 (d, *J =* 7.2 Hz, 2H), 2.18–2.00 (m, 5H), 1.92 (dd, *J =* 15.3 Hz, 7.3 Hz, 2H), 1.73 (d, *J =* 1.0 Hz, 3H), 1.67 (s, 3H), 1.58 (s, 3H), 1.26 (dd, *J =* 15.4 Hz, 7.3 Hz, 2H), 0.94 (d, *J =* 6.7 Hz, 3H); Isomer (2*E*,6*E*) δ: 5.44–5.38 (m, 1H), 5.34–5.18 (m, 2H), 5.12–5.04 (m, 1H), 4.12 (d, *J =* 7.1 Hz, 2H), 2.15–2.00 (m, 5H), 1.92 (dd, *J =* 15.3 Hz, 7.2 Hz, 2H), 1.67 (s, 3H), 1.66 (d, *J =* 0.9 Hz, 3H), 1.58 (s, 3H), 1.25 (dd, *J =* 14.8 Hz, 7.2 Hz, 2H), 0.94 (d, *J =* 6.7 Hz, 3H); ^13^C-NMR (63 MHz, CDCl_3_) Isomer (2*Z*,6*E*) δ: 138.7 (s), 136.7 (d), 130.8 (s), 127.5 (d), 124.6 (d), 124.5 (d), 58.6 (t), 37.0 (t), 36.1 (d), 32.0 (t), 31.0 (t), 25.6 (t), 25.5 (q), 23.2 (q), 20.6 (q), 17.4 (q); Isomer (2*E*,6*E*) δ: 138.5 (s), 136.4 (d), 130.8 (s), 127.7 (d), 124.6 (d), 123.6 (d), 58.8 (t), 39.5 (t), 37.0 (t), 36.1 (d), 30.7 (t), 25.6 (t), 25.5 (q), 20.7 (q), 17.4 (q), 16.0 (q); MS (70 eV) *m*/*z* (%) Isomer (2*Z*,6*E*) 236 [M]^+^ (0), 218 ([M]^+^-18, 1), 69 (100), 41 (51), 82 (47), 81 (45), 55 (36), 95 (34), 67 (30), 93 (26), 107 (21), 109 (17); Isomer (2*E*,6*E*) 236 [M]^+^ (0), 218 ([M]^+^-18, 1), 69 (100), 82 (66), 41 (59), 81 (58), 95 (50), 55 (42), 67 (38), 93 (27), 107 (26), 109 (24), 135 (14). Anal. Calcd for: C_16_H_28_O: C, 81.29; H, 11.94. Found: C, 81.12; H, 11.89.

#### 3.3.3. 3,7,8,12-Tetramethyltrideca-2,6-dien-1-ol (**26**)

Yield 95%. IR (ATR) 3309, 2955, 2925, 2869, 1667, 1456, 1380, 1368, 1092, 1002, 955, 836 cm^−1^. ^1^H-NMR (250 MHz, CDCl_3_) Isomer (2*Z*,6*Z*) and (2*Z*,6*E*) δ: 5.48–5.38 (m, 1H), 5.14–5.04 (m, 1H), 4.11 (d, *J =* 7.1 Hz, 2H), 2.13–2.00 (m, 5H), 1.75 (d, *J =* 1.0 Hz, 3H), 1.59–1.46 (m, 1H), 1.51 (d, *J =* 1.2 Hz, 3H), 1.35–1.10 (m, 6H), 0.95 (d, *J =* 6.9 Hz, 3H), 0.86 (d, *J =* 6.6 Hz, 6H); Isomer (2*E*,6*Z*) and (2*E*,6*E*) δ: 5.46–5.38 (m, 1H), 5.10 (t, *J =* 5.7 Hz, 1H), 4.15 (d, *J =* 7.0 Hz, 2H), 2.18–1.97 (m, 5H), 1.68 (d, *J =* 0.6 Hz, 3H), 1.62–1.44 (m, 1H), 1.50 (d, *J =* 1.2 Hz, 3H), 1.33–1.05 (m, 6H), 0.95 (d, *J =* 6.9 Hz, 3H), 0.85 (d, *J =* 6.6 Hz, 3H), 0.84 (d, *J =* 6.6 Hz, 3H); ^13^C-NMR (63 MHz, CDCl_3_) Isomer (2*Z*,6*Z*) δ: 140.3 (s), 139.7 (s), 124.5 (d), 124.2 (d), 58.9 (t), 39.2 (t), 35.0 (t), 33.9 (d), 32.3 (t), 27.9 (d), 25.8 (t), 25.5 (t), 23.4 (q), 22.7 (q), 22.6 (q), 19.4 (q), 17.9 (q); Isomer (2*Z*,6*E*) δ: 140.2 (s), 139.9 (s), 124.3 (d), 122.7 (d), 59.0 (t), 42.7 (d), 39.1 (t), 35.1 (t), 32.0 (t), 27.9 (d), 26.3 (t), 25.3 (t), 23.4 (q), 22.7 (q), 22.6 (q), 19.7 (q), 12.2 (q); Isomer (2*E*,6*Z*) δ: 139.7 (s), 139.6 (s), 124.2 (d), 123.3 (d), 62.3 (t), 39.9 (t), 39.2 (t), 35.0 (t), 33.9 (d), 27.9 (d), 25.6 (t), 25.5 (t), 22.6 (q), 22.6 (q), 19.4 (q), 17.9 (q), 16.2 (q); Isomer (2*E*,6*E*) δ: 149.7 (s), 139.6 (s), 123.3 (d), 123.0 (d), 62.3 (t), 42.7 (d), 39.6 (t), 39.1 (t), 35.1 (t), 27.9 (d), 26.1 (t), 25.2 (t), 22.7 (q), 22.6 (q), 19.8 (q), 16.2 (q), 12.1 (q); MS (70 eV) *m*/*z* (%) Isomer (2*Z*,6*Z*) 252 [M]^+^ (4), 69 (100), 55 (74), 111 (44), 83 (41), 84 (39), 97 (37), 57 (33), 93 (32), 41 (30), 43 (28), 121 (24), 221 (4); Isomer (2*Z*,6*E*) 252 [M]^+^ (3), 69 (100), 55 (67), 111 (54), 83 (36), 97 (35), 57 (34), 41 (28), 43 (25), 81 (25), 93 (24), 121 (15), 221 (4); Isomer (2*E*,6*Z*) 252 [M]^+^ (3), 69 (100), 55 (75), 111 (50), 83 (41), 97 (40), 57 (36), 84 (33), 93 (31), 41 (31), 43 (28), 121 (22), 221 (3); Isomer (2*E*,6*E*) 252 [M]^+^ (2), 69 (100), 55 (61), 111 (53), 83 (34), 97 (32), 57 (31), 41 (24), 43 (21), 81 (21), 93 (18), 121 (11), 221 (3). Anal. Calcd for: C_17_H_32_O: C, 80.89; H, 12.78. Found: C, 81.03; H, 12.85.

#### 3.3.4. 3,5,9-Trimethyldeca-2,8-dien-1-ol (**27**)

Boiling point 94–96 °C/0.5 Torr. Yield 69%. IR (ATR) 3312, 2964, 2913, 2872, 1668, 1449, 1377, 1063, 996, 889 cm^−1^; ^1^H-NMR (250 MHz, CDCl_3_) Isomer *Z*: δ: 5.42–5.34 (m, 1H), 5.14–5.04 (m, 1H), 4.15 (d, *J =* 6.9 Hz, 2H), 2.08–1.97 (m, 4H), 1.70 (d, *J =* 1.2 Hz, 3H), 1.68 (d, *J =* 1.1 Hz, 3H), 1.65–1.52 (m, 1H), 1.60 (s, 3H), 1.40–1.24 (m, 1H), 1.18–1.04 (m, 1H), 0.82 (d, *J =* 6.6 Hz, 3H); Isomer *E*: δ: 5.52–5.43 (m, 1H), 5.14–5.04 (m, 1H), 4.11 (d, *J =* 7.1 Hz, 2H), 2.09–1.99 (m, 2H), 1.92 (dd, *J =* 13.1 Hz , 8.3 Hz, 1H), 1.78 (dd, *J =* 13.1 Hz, 8.3 Hz, 1H), 1.68 (d, *J =* 1.1 Hz, 3H), 1.65–1.52 (m, 1H), 1.64 (s, 3H), 1.60 (s, 3H), 1.40–1.24 (m, 1H), 1.18–1.04 (m, 1H), 0.83 (d, *J =* 6.5 Hz, 3H); ^13^C-NMR (63 MHz, CDCl_3_) Isomer *Z*: δ: 137.7 (s), 130.8 (s), 125.5 (d), 125.0 (d), 58.6 (t), 39.3 (t), 36.8 (t), 30.6 (d), 25.4 (q), 25.3 (t), 23.4 (q), 19.1 (q), 17.3 (q); Isomer *E*: δ: 137.4 (s), 130.7 (s), 124.6 (d), 124.5 (d), 58.7 (t), 47.4 (t), 36.7 (t), 30.0 (d), 25.4 (q), 25.3 (t), 19.1 (q), 17.3 (q), 15.7 (q); MS (70 eV) *m*/*z* (%) Isomer *Z*: 196 [M]^+^ (2), 69 (100), 109 (66), 41 (63), 95 (62), 55 (46), 111 (42), 82 (42), 67 (40), 107 (36), 81 (28), 93 (25), 163 (15); Isomer *E*: 196 [M]^+^ (2), 69 (100), 41 (65), 95 (64), 109 (63), 82 (55), 55 (41), 67 (38), 81 (26), 107 (23), 111 (20). Anal. Calcd for: C_13_H_24_O: C, 79.53; H, 12.32. Found: C, 79.44; H, 12.36.

#### 3.3.5. 3,6,10-Trimethylundeca-2,9-dien-1-ol (**28**)

Yield 97%. IR (ATR) 3319, 2963, 2915, 2855, 1669, 1452, 1377, 1114, 1075, 998, 954 cm^−1^; ^1^H-NMR (700 MHz, CDCl_3_) Isomer *Z*: δ: 5.39 (t, *J =* 6.9 Hz, 1H), 5.09 (t, *J =* 6.9 Hz, 1H), 4.12 (d, *J =* 6.9 Hz, 1H), 2.10*–*2.01 (m, 2H), 1.99 (dd, *J =* 14.8, 6.9 Hz, 1H), 1.96*–*1.89 (m, 1H), 1.73 (s, 3H), 1.68 (s, 3H), 1.60 (s, 3H), 1.40 (dd, *J =* 12.3, 6.5 Hz, 1H), 1.38*–*1.35 (m, 1H), 1.34*–*1.30 (m, 1H), 1.21*–*1.18 (m, 1H), 1.17*–*1.12 (m, 1H), 0.89 (d, *J =* 6.5 Hz, 3H); Isomer *E*: δ: 5.43*–*5.39 (m, 1H), 5.12*–*5.08 (m, 1H), 4.14 (s. 2H), 2.07*–*2.02 (m, 1H), 2.01*–*1.96 (m, 2H), 1.93 (dd, *J =* 14.5, 7.1 Hz, 1H), 1.68 (d, *J =* 1.2 Hz, 3H), 1.67 (s, 3H), 1.60 (s, 3H), 1.46*–*1.41 (m, 1H), 1.39 (dd, *J =* 12.8, 6.6 Hz, 1H), 1.36*–*1.31 (m, 1H), 1.26*–*1.20 (m, 1H), 1.18*–*1.12 (m, 1H), 0.88 (d, *J =* 6.6 Hz, 3H); ^13^C-NMR (176 MHz, CDCl_3_) Isomer *Z*: δ: 140.4 (s), 131.0 (s), 124.7 (d), 123.8 (d), 58.8 (t), 36.8 (t), 35.6 (t), 32.4 (d), 29.4 (t), 25.6 (q), 25.4 (t), 23.4 (q), 19.4 (q), 17.5 (q); Isomer *E*: δ: 140.0 (s), 130.9 (s), 124.8 (d), 123.0 (d), 59.2 (t), 37.0 (t), 36.9 (t), 34.9 (t), 32.1 (d), 25.6 (q), 25.4 (t), 19.4 (q), 17.5 (q), 16.1 (q); MS (70 eV) *m*/*z* (%) Isomer *Z*: 210 [M]^+^ (3), 69 (100), 109 (65), 81 (59), 41 (59), 55 (44), 82 (43), 107 (37), 67 (35), 84 (33), 177 (12); Isomer *E*: 210 [M]^+^ (1), 69 (100), 109 (77), 81 (67), 41 (58), 82 (40), 55 (40), 67 (32), 95 (27), 71 (25), 107 (22). Anal. Calcd for: C_14_H_26_O: C, 79.94; H, 12.46. Found: C, 80.11; H, 12.53.

#### 3.3.6. 3,4,6,10-Tetramethylundeca-2,9-dien-1-ol (**29**)

Yield 49%. IR (ATR) 3322, 2963, 2914, 2872, 1664, 1455, 1377, 1161, 1129, 1028, 998, 953, 818 cm^−1^; ^1^H-NMR (250 MHz, CDCl_3_) *Z* Isomers δ: 5.35 (td, *J =* 6.9 Hz, 1.3 Hz, 1H), 5.12-5.02 (m, 1H), 4.18–4.11 (m, 2H), 2.75 (dd, *J =* 14.3 Hz, 6.8 Hz, 1H), 2.05–1.85 (m, 2H), 1.67 (d, *J =* 1.4 Hz, 3H), 1.62 (d, *J =* 1.3 Hz, 3H), 1.59 (s, 3H), 1.42–1.10 (m, 5H), Racemate 2: 0.97 (d, *J =* 6.8 Hz, 3H), Racemate 1: 0.94 (d, *J =* 6.8 Hz, 3H), 0.86 (d, *J =* 6.2 Hz, 3H); *E* Isomers δ: 5.41 (td, *J =* 6.8 Hz, 0.6 Hz, 1H), 5.13–5.03 (m, 1H), 4.14 (d, *J =* 6.8 Hz, 2H), 2.31–2.16 (m, 1H), 2.02–1.88 (m, 2H), 1.67 (s, 3H), 1.59 (s, 3H), 1.56 (d, *J =* 0.6 Hz, 3H), 1.42–1.10 (m, 5H), Racemate 3: 0.97 (d, *J =* 6.8 Hz, 3H), Racemate 4: 0.95 (d, *J =* 6.8 Hz, 3H), 0.84 (d, *J =* 6.2 Hz, 3H); ^13^C-NMR (63 MHz, CDCl_3_) Racemate 1 (*Z*) δ: 144.3 (s), 131.1 (s), 124.8 (d), 123.8 (d), 58.5 (t), 42.4 (t), 37.0 (t), 31.6 (d), 30.1 (d), 25.6 (q), 25.4 (t), 19.9 (q), 19.2 (q), 18.1 (q), 17.6 (q); Racemate 2 (*Z*) δ: 143.5 (s), 131.0 (s), 124.8 (d), 124.4 (d), 58.6 (t), 41.9 (t), 37.8 (t), 31.6 (d), 30.2 (d), 25.6 (q), 25.4 (t), 20.1 (q), 19.6 (q), 17.8 (q), 17.6 (q); Racemate 3 (*E*) δ: 143.5 (s), 131.0 (s), 124.9 (d), 123.2 (d), 59.2 (t), 42.1 (t), 40.0 (d), 37.5 (t), 30.0 (d), 25.6 (q), 25.4 (t), 20.2 (q), 19.6 (q), 17.6 (q), 11.9 (q); Racemate 4 (*E*) δ: 144.3 (s), 131.0 (s), 124.9 (d), 123.6 (d), 59.2 (t), 42.5 (t), 39.8 (d), 37.0 (t), 29.9 (d), 25.6 (q), 25.3 (t), 19.7 (q), 19.2 (q), 17.6 (q), 12.8 (q); MS (70 eV) *m*/*z* (%) Racemate 1 and 2 (*Z*) 224 [M]^+^ (2), 69 (100), 82 (77), 109 (65), 81 (55), 41 (55), 95 (44), 55 (44), 67 (40) 98 (39), 43 (33) 123 (20), 135 (11), 151 (10), 191 (6); Racemate 3 and 4 (*E*) 224 [M]^+^ (4), 69 (100), 82 (71), 109 (61), 81 (60), 41 (50), 55 (40), 95 (39), 67 (37), 43 (33), 83 (24), 71 (19), 123 (16), 151 (15), 191 (6). Anal. Calcd for: C_15_H_28_O: C, 80.29; H, 12.58. Found: C, 80.17; H, 12.63.

### 3.4. Preparation of Compounds Structurally Related to Farnesyl Acetate *(**30**–**35**)*

To the stirred and cooled (*ca.* 0 °C) solution of 6.7 mmol of appropriate compound structurally related to farnesol (**24**–**29**) and 0.82 g (8 mmol) of acetic anhydride, 0.81 g (8 mmol) of triethylamine was added. After addition, the mixture was stirred for 2 h. Next, 2 mL of methanol was added and stirring was continued for another 0.5 h. Afterwards, 30 mL of hexane and 20 mL of water was added and the hexane layer was separated, washed with brine and dried over anhydrous magnesium sulphate. The obtained acetates were purified by column chromatography on silica with hexane: ethyl acetate (98:2) as the mobile phase. Purity of the products according to GC-MS was 92%–99%.

#### 3.4.1. 3,7,8,12-Tetramethyltrideca-2,6,11-trien-1-yl acetate (**30**)

Yield 90%. IR (ATR) 2964, 2923, 2857, 1740, 1666, 1450, 1377, 1230, 1022, 953 cm^−1^. ^1^H-NMR (250 MHz, CDCl_3_) Isomer (2*Z*,6*Z*) and (2*Z*,6*E*) δ: 5.40*–*5.31 (m, 1H), 5.14*–*5.04 (m, 2H), 4.56 (d, *J =* 7.2 Hz, 2H), 2.15*–*2.02 (m, 5H), 2.05 (s, 3H), 1.85 (dd, *J =* 14.7 Hz, 7.3 Hz, 2H), 1.77 (d, *J =* 1.1 Hz, 3H), 1.67 (s, 3H), 1.58 (s, 3H), 1.51 (d, *J =* 1.0 Hz, 3H), 1.43*–*1.18 (m, 2H), 0.95 (d, *J =* 6.9 Hz, 3H); Isomer (2*E*,6*Z*) and (2*E*,6*E*) δ: 5.38*–*5.30 (m, 1H), 5.13*–*5.05 (m, 2H), 4.58 (d, *J =* 7.1 Hz, 2H), 2.17*–*2.00 (m, 5H), 2.05 (s, 3H), 1.85 (dd, *J =* 14.7 Hz, 7.1 Hz, 3H), 1.70 (s, 3H), 1.67 (s, 3H), 1.58 (s, 3H), 1.50 (d, *J =* 0.8 Hz, 3H), 1.44*–*1.16 (m, 2H), 0.95 (d, *J =* 6.9 Hz, 3H); ^13^C-NMR (63 MHz, CDCl_3_) Isomer (2*Z*,6*Z*) δ: 170.8 (s), 142.3 (s), 139.6 (s), 131.0 (s), 124.7 (d), 124.1 (d), 119.1 (d), 60.9 (t), 34.8 (t), 33.4 (d), 32.4 (t), 26.1 (t), 25.8 (t), 25.5 (q), 23.4 (q), 20.8 (q), 19.1 (q), 17.8 (q), 17.5 (q); Isomer (2*Z*,6*E*) δ: 170.8 (s), 142.4 (s), 139.7 (s), 130.8 (s), 124.8 (d), 122.8 (d), 119.1 (d), 60.9 (t), 42.3 (d), 35.0 (t), 32.0 (t), 26.3 (t), 26.0 (t), 25.5 (q), 23.3 (q), 20.8 (q), 19.6 (q), 17.5 (q), 12.1 (q); Isomer (2*E*,6*Z*) δ: 170.8 (s), 141.9 (s), 139.3 (s), 131.0 (s), 124.7 (d), 124.3 (d), 118.2 (d), 61.2 (t), 39.8 (t), 34.8 (t), 33.4 (d), 26.1 (t), 25.5 (q), 25.4 (t), 20.8 (q), 19.1 (q), 17.8 (q), 17.5 (q), 16.3 (q); Isomer (2*E*,6*E*) δ: 170.8 (s), 141.9 (s), 139.3 (s), 130.8 (s), 124.8 (d), 123.1 (d), 118.3 (d), 61.2 (t), 42.2 (d), 39.4 (t), 34.9 (t), 26.0 (t), 25.8 (t), 25.5 (q), 20.8 (q), 19.7 (q), 17.5 (q), 16.2 (q), 12.1 (q); MS (70 eV) *m*/*z* (%) Isomer (2*Z*,6*Z*) 292 [M]^+^ (0), 82 (100), 69 (56), 109 (32), 95 (27), 41 (24), 55 (24), 67 (24), 81 (24), 43 (21), 83 (20), 121 (15); Isomer (2*Z*,6*E*) and (2*E*,6*Z*) 292 [M]^+^ (0), 82 (100), 69 (57), 109 (30), 95 (27), 67 (23), 81 (23), 41 (22), 55 (22), 43 (21), 83 (20), 121 (12); Isomer (2*E*,6*E*) 292 [M]^+^ (0), 82 (100), 69 (52), 109 (29), 95 (26), 43 (23), 67 (22), 81 (21), 41 (19), 55 (18), 83 (17), 121 (11). Anal. Calcd for: C_19_H_32_O_2_: C, 78.03; H, 11.03. Found: C, 77.87; H, 11.08.

#### 3.4.2. (*E*)-3,8,12-Trimethyltrideca-2,6,11-trien-1-yl acetate (**31**)

Yield 83%. IR (ATR) 2963, 2916, 2854, 1740, 1671, 1450, 1376, 1230, 1022, 968 cm^−1^; ^1^H-NMR (250 MHz, CDCl_3_) Isomer (2*Z*,6*E*) δ: 5.39*–*5.18 (m, 3H), 5.12*–*5.02 (m, 1H), 4.54 (d, *J =* 7.2 Hz, 2H), 2.19*–*1.97 (m, 5H), 1.92 (dd, *J =* 15.1 Hz, 7.3 Hz, 2H), 1.74 (d, *J =* 1.3 Hz, 3H), 1.66 (s, 3H), 1.57 (s, 3H), 1.25 (dd, *J =* 15.4 Hz, 7.3 Hz, 2H), 0.94 (d, *J =* 6.7 Hz, 3H); Isomer (2*E*,6*E*) δ: 5.40*–*5.18 (m, 3H), 5.12*–*5.03 (m, 1H), 4.56 (d, *J =* 7.0 Hz, 2H), 2.18*–*1.98 (m, 5H), 1.91 (dd, *J =* 15.1 Hz, 7.1 Hz, 2H), 1.68 (s, 3H), 1.67 (d, *J =* 0.9 Hz, 3H), 1.57 (s, 3H), 1.25 (dd, *J =* 15.4 Hz, 7.1 Hz, 2H), 0.93 (d, *J =* 6.7 Hz, 3H); ^13^C-NMR (63 MHz, CDCl_3_) Isomer (2*Z*,6*E*) δ: 169.9 (s), 141.0 (s), 136.4 (d), 130.2 (s), 126.9 (d), 124.3 (d), 119.2 (d), 60.3 (t), 36.7 (t), 35.8 (d), 31.7 (t), 30.6 (t), 25.3 (t), 25.1 (q), 22.8 (q), 20.2 (q), 20.1 (q), 17.0 (q); Isomer (2*E*,6*E*) δ: 169.8 (s), 140.7 (s), 136.2 (d), 130.1 (s), 127.2 (d), 124.3 (d), 118.4 (d), 60.5 (t), 39.1 (t), 36.7 (t), 35.8 (d), 30.2 (t), 25.3 (t), 25.1 (q), 20.4 (q), 20.1 (q), 17.0 (q), 15.7 (q); MS (70 eV) *m*/*z* (%) Isomer (2*Z*,6*E*) 278 [M]^+^ (1), 69 (100), 82 (70), 43 (65), 41 (59), 95 (54), 81 (53), 94 (52), 55 (48), 93 (46), 67 (45), 107 (40), 175 (15), 203 (7), 218 (5); Isomer (2*E*,6*E*) 278 [M]^+^ (1), 69 (100), 43 (83), 82 (66), 41 (62), 95 (56), 81 (53), 67 (44), 94 (43), 55 (42), 93 (41), 107 (40), 150 (20), 203 (10), 218 (4). Anal. Calcd for: C_18_H_30_O_2_: C, 77.65; H, 10.86. Found: C, 77.48; H, 10.97.

#### 3.4.3. 3,7,8,12-Tetramethyltrideca-2,6-dien-1-yl acetate (**32**)

Yield 69%. IR (ATR) 2956, 2926, 2869, 1741, 1665, 1455, 1378, 1366, 1230, 1022, 954 cm^−1^; ^1^H-NMR (250 MHz, CDCl_3_) Isomer (2*Z*,6*Z*) and (2*Z*,6*E*) δ: 5.37*–*5.27 (m, 1H), 5.12*–*5.01 (m, 1H), 4.54 (d, *J =* 7.2 Hz, 2H), 2.11*–*1.98 (m, 5H), 2.01 (s, 3H), 1.73 (s, 3H), 1.55*–*1.40 (m, 1H), 1.47 (s, 3H), 1.32*–*1.06 (m, 6H), 0.92 (d, *J =* 6.9 Hz, 3H), 0.82 (d, *J =* 6.6 Hz, 6H); Isomer (2*E*,6*Z*) and (2*E*,6*E*) δ: 5.36*–*5.27 (m, 1H), 5.10*–*5.00 (m, 1H), 4.56 (d, *J =* 7.1 Hz, 2H), 2.15*–*1.95 (m, 5H), 2.03 (s, 3H), 1.68 (s, 3H), 1.56*–*1.41 (m, 1H), 1.47 (d, *J =* 1.1 Hz, 3H), 1.32*–*1.05 (m, 6H), 0.92 (d, *J =* 6.6 Hz, 3H), 0.83 (d, *J =* 6.6 Hz, 3H), 0.82 (d, *J =* 6.6 Hz, 3H); ^13^C-NMR (63 MHz, CDCl_3_) Isomer (2*Z*,6*Z*) δ: 170.9 (s), 142.4 (s), 139.9 (s), 123.9 (d), 119.2 (d), 61.0 (t), 39.2 (t), 35.0 (t), 33.9 (d), 32.5 (t), 27.8 (d), 25.9 (t), 25.5 (t), 23.4 (q), 22.6 (q), 22.5 (q), 20.9 (q), 19.3 (q), 17.9 (q); Isomer (2*Z*,6*E*) δ: 170.9 (s), 142.5 (s), 140.0 (s), 122.6 (d), 119.1 (d), 61.0 (t), 42.6 (d), 39.1 (t), 35.1 (t), 32.1 (t), 27.9 (d), 26.3 (t), 25.3 (t), 23.4 (q), 22.6 (q), 22.5 (q), 20.9 (q), 19.7 (q), 12.2 (q); Isomer (2*E*,6*Z*) δ: 170.9 (s), 142.1 (s), 139.7 (s), 124.1 (d), 118.3 (d), 61.3 (t), 39.9 (t), 39.2 (t), 35.0 (t), 33.9 (d), 27.9 (d), 25.5 (t), 25.5 (t), 22.6 (q), 22.5 (q), 20.9 (q), 19.3 (q), 17.9 (q), 16.3 (q); Isomer (2*E*,6*E*) δ: 170.9 (s), 142.1 (s), 139.7 (s), 122.8 (d), 118.3 (d), 61.3 (t), 42.6 (d), 39.5 (t), 39.1 (t), 35.1 (t), 27.9 (d), 25.9 (t), 25.3 (t), 22.6 (q), 22.5 (q), 20.9 (q), 19.7 (q), 16.3 (q), 12.2 (q); MS (70 eV) *m*/*z* (%) Isomer (2*Z*,6*Z*) 294 [M]^+^ (0), 69 (100), 55 (64), 80 (53), 93 (52), 43 (51), 121 (43), 111 (34), 83 (33), 97 (32), 149 (13), 234 (8), 166 (7); Isomer (2*Z*,6*E*) and (2*E*,6*Z*) 294 [M]^+^ (0), 69 (100), 55 (63), 43 (49), 93 (44), 80 (44), 111 (42), 121 (35), 97 (35), 83 (34), 57 (33), 149 (15), 166 (9), 234 (8); Isomer (2*E*,6*E*) 294 [M]^+^ (0), 69 (100), 55 (61), 43 (52), 111 (46), 93 (34), 83 (33), 97 (32), 57 (32), 121 (24), 149 (14), 166 (14), 234 (6). Anal. Calcd for: C_19_H_34_O_2_: C, 77.50; H, 11.64. Found: C, 77.38; H, 11.73.

#### 3.4.4. 3,5,9-Trimethyldeca-2,8-dien-1-yl acetate (**33**)

Yield 82%. IR (ATR) 2966, 2916, 2849, 1740, 1668, 1453, 1378, 1229, 1022, 953 cm^−1^; ^1^H-NMR (700 MHz, CDCl_3_) Isomer *Z*: δ: 5.41 (t, *J =* 7.1 Hz, 1H), 5.10*–*5.06 (m, 1H), 4.56 (d, *J =* 7.1 Hz, 2H), 2.06*–*2.00 (m, 2H), 2.04 (s, 3H), 1.95 (dd, *J =* 13.3 Hz, 8.5 Hz, 2H), 1.73 (s, 3H), 1.68 (s, 3H), 1.63 (dd, *J =* 13.3 Hz, 6.5 Hz, 1H), 1.60 (s, 3H), 1.34*–*1.28 (m, 1H), 1.16–1.09 (m, 2H), 0.83 (d, *J =* 6.5 Hz, 3H); Isomer *E*: δ: 5.33*–*5.30 (m, 1H), 5.10*–*5.06 (m, 1H), 4.59 (d, *J =* 7.1 Hz, 2H), 2.05 (s, 3H), 2.01 (dd, *J =* 15.4 Hz, 7.1 Hz, 2H), 1.95 (dd, *J =* 13.3 Hz, 8.4 Hz, 1H), 1.80 (dd, *J =* 13.3 Hz, 8.4 Hz, 1H), 1.68 (s, 3H), 1.66 (s, 3H), 1.63 (dd, *J =* 13.3 Hz, 6.6 Hz, 1H), 1.60 (s, 3H), 1.34*–*1.28 (m, 1H), 1.15–1.08 (m, 2H), 0.82 (d, *J =* 6.6 Hz, 3H); ^13^C-NMR (176 MHz, CDCl_3_) Isomer *Z*: δ: 171.0 (s), 141.7 (s), 131.1 (s), 124.6 (d), 120.0 (d), 61.1 (t), 39.5 (t), 36.9 (t), 30.8 (d), 25.6 (q), 25.6 (t), 23.6 (q), 20.9 (q), 19.2 (q), 17.6 (q); Isomer *E*: δ: 171.0 (s), 141.2 (s), 131.1 (s), 124.7 (d), 119.7 (d), 61.3 (t), 47.5 (t), 36.8 (t), 30.1 (d), 25.6 (q), 25.4 (t), 20.9 (q), 19.3 (q), 17.5 (q), 16.1 (q); MS (70 eV) *m*/*z* (%) Isomer *Z*: 238 [M]^+^ (1), 69 (100), 95 (84), 109 (78), 107 (69), 43 (58), 41 (54), 82 (43), 55 (43), 67 (42), 163 (41), 93 (40), 81 (38), 122 (25), 135 (23), 178 (16); Isomer *E*: 238 [M]^+^ (1), 69 (100), 95 (81), 109 (71), 107 (66), 43 (60), 41 (50), 82 (46), 67 (42), 55 (39), 163 (38), 93 (38), 81 (33), 122 (22), 135 (20), 178 (16). Anal. Calcd for: C_15_H_26_O_2_: C, 75.58; H, 10.99. Found: C, 75.77; H, 10.83.

#### 3.4.5. 3,6,10-Trimethylundeca-2,9-dien-1-yl acetate (**34**)

Yield 86%. IR (ATR) 2965, 2916, 2856, 1740, 1671, 1453, 1378, 1229, 1122, 853 cm^−1^; ^1^H-NMR (700 MHz, CDCl_3_) Isomer *Z*: δ: 5.34*–*5.31 (m, 1H), 5.11*–*5.08 (m, 1H), 4.56 (d, *J =* 7.1 Hz, 1H), 2.10*–*2.02 (m, 2H), 2.05 (s, 3H), 2.02*–*1.97 (m, 1H), 1.97*–*1.91 (m, 1H), 1.75 (s, 3H), 1.68 (s, 3H), 1.60 (s, 3H), 1.46*–*1.36 (m, 2H), 1.35*–*1.30 (m, 1H), 1.26*–*1.18 (m, 1H), 1.18*–*1.12 (m, 1H), 0.90 (d, *J =* 6.5 Hz, 3H); Isomer *E*: δ: 5.36*–*5.31 (m, 1H), 5.11*–*5.08 (m, 1H), 4.58 (d, *J =* 7.1 Hz, 1H), 2.09*–*2.03 (m, 1H), 2.05 (s, 3H), 2.02*–*1.97 (m, 2H), 1.96*–*1.90 (m, 1H), 1.69 (s, 3H), 1.68 (s, 3H), 1.60 (s, 3H), 1.46*–*1.41 (m, 1H), 1.39 (dd, *J =* 12.6, 6.5 Hz, 1H), 1.36*–*1.30 (m, 1H), 1.26*–*1.20 (m, 1H), 1.18*–*1.12 (m, 1H), 0.88 (d, *J =* 6.5 Hz, 3H); ^13^C-NMR (176 MHz, CDCl_3_) Isomer *Z*: δ: 170.9 (s), 143.4 (s), 131.0 (s), 124.7 (d), 118.5 (d), 60.9 (t), 36.9 (t), 35.4 (t), 32.4 (d), 29.6 (t), 25.6 (q), 25.4 (t), 23.4 (q), 20.9 (q), 19.4 (q), 17.5 (q); Isomer *E*: δ: 170.9 (s), 142.8 (s), 130.9 (s), 124.8 (d), 117.9 (d), 61.3 (t), 37.0 (t), 36.9 (t), 34.8 (t), 32.1 (d), 25.6 (q), 25.4 (t), 20.9 (q), 19.4 (q), 17.5 (q), 16.3 (q); MS (70 eV) *m*/*z* (%) Isomer *Z*: 252 [M]^+^ (1), 69 (100), 81 (72), 109 (68), 107 (60), 41 (52), 43 (51), 82 (50), 67 (39), 55 (36), 93 (31), 121 (28), 136 (25), 177 (23), 149 (20); Isomer *E*: 252 [M]^+^ (2), 69 (100), 81 (77), 109 (67), 43 (56), 107 (54), 41 (51), 82 (45), 67 (36), 55 (34), 93 (28), 121 (26), 136 (20), 149 (19), 177 (18). Anal. Calcd for: C_16_H_28_O_2_: C, 76.14; H, 11.18. Found: C, 76.31; H, 11.23.

#### 3.4.6. 3,4,6,10-Tetramethylundec-2,9-dien-1-yl acetate (**35**)

Yield 91%. IR (ATR) 2956, 2915, 2869, 1741, 1665, 1455, 1375, 1230, 1022, 953 cm^−1^; ^1^H-NMR (250 MHz, CDCl_3_) Racemate 1 and 2 (*Z*) δ: 5.34*–*5.25 (m, 1H), 5.12*–*5.02 (m, 1H), 4.62*–*4.54 (m, 2H), 2.83*–*2.71 (m, 1H), 2.03 (s, 3H), 2.00*–*1.86 (m, 2H), 1.67 (s, 3H), 1.64 (d, *J =* 1.4 Hz, 3H), 1.59 (s, 3H), 1.47*–*1.06 (m, 5H), Racemate 2: 0.96 (d, *J =* 6.9 Hz, 3H), Racemate 1: 0.94 (d, *J =* 6.9 Hz, 3H), 0.85 (t, *J =* 6.4 Hz, 3H); Racemate 3 and 4 (*E*) δ: 5.34 (t, *J =* 6.9 Hz, 1H), 5.12*–*5.02 (m, 1H), 4.58 (d, *J =* 6.9 Hz, 2H), 2.33*–*2.18 (m, 1H), 2.04 (s, 3H), 2.00*–*1.86 (m, 2H), 1.67 (s, 3H), 1.61 (d, *J =* 0.6 Hz, 3H), 1.59 (s, 3H), 1.47*–*1.06 (m, 5H), Racemate 3: 0.97 (d, *J =* 6.9 Hz, 3H), 0.84 (d, *J =* 6.3 Hz, 3H Racemate 4: 0.96 (d, *J =* 6.9 Hz, 3H), 0.84 (d, *J =* 6.9 Hz, 3H); ^13^C-NMR (63 MHz, CDCl_3_) Racemate 1 and 2: 170.9 (s), 147.0 (s), 146.3 (s), 130.8 (s), 124.8 (d), 119.0 (d), 118.5 (d), 60.4 (t), 42.2 (t), 41.8 (t), 37.7 (t), 31.7 (d), 30.1 (d), 30.0 (d), 25.6 (q), 25.3 (t), 20.9 (q), 19.9 (q), 19.8 (q), 19.6 (q), 18.9 (q), 18.1 (q), 17.8 (q), 17.5 (q); Racemate 3: 170.8 (s), 145.7 (s), 130.8 (s), 124.8 (d), 118.0 (d), 61.2 (t), 42.0 (t), 40.0 (d), 37.4 (t), 30.0 (d), 25.6 (q), 25.3 (t), 20.9 (q), 20.0 (q), 19.5 (q), 17.5 (q), 12.1 (q); Racemate 4: 170.9 (s), 146.6 (s), 130.8 (s), 124.8 (d), 117.5 (d), 61.3 (t), 42.4 (t), 39.8 (d), 37.0 (t), 29.9 (d), 25.6 (q), 25.3 (t), 20.9 (q), 19.7 (q), 19.1 (q), 17.5 (q), 12.8 (q); MS (70 eV) *m*/*z* (%) Racemate 1 and 2 (*Z*) 266 [M]^+^ (1), 82 (100), 69 (76), 43 (57), 109 (55), 81 (52), 95 (44), 67 (38), 41 (37), 55 (32), 107 (28), 121 (26), 123 (25), 135 (15), 191 (12), 206 (6); Racemate 3 (*E*) 266 [M]^+^ (2), 82 (100), 69 (94), 43 (71), 109 (65), 81 (54), 95 (53), 41 (47), 67 (40), 121 (36), 55 (33), 107 (31), 123 (27), 135 (20), 191 (12), 206 (7); Racemate 4 (*E*) 266 [M]^+^ (1), 69 (100), 82 (97), 43 (71), 81 (67), 109 (65), 95 (53), 41 (47), 67 (40), 121 (38), 55 (34), 107 (30), 123 (26), 135 (19), 191 (11), 206 (6). Anal. Calcd for: C_17_H_30_O_2_: C, 76.41; H, 11.35. Found: C, 76.49; H, 11.28.

## 4. Conclusions

The compounds described in this paper are potentially useful in fragrance compositions as antimicrobial agents and showed better or comparable activity to parent terpenoids. Their negligible toxicity to the tested human cell lines may indicate possible safe use. The generated pharmacophore models indicate significant steric factors which determine the antimicrobial activity of the compounds described here and probably of other structurally similar compounds.
